# Hydride-Free Hydrogenation: Unraveling the Mechanism of Electrocatalytic Alkyne Semihydrogenation by Nickel–Bipyridine Complexes

**DOI:** 10.1021/jacs.3c03340

**Published:** 2023-07-25

**Authors:** Gabriel Durin, Mi-Young Lee, Martina A. Pogany, Thomas Weyhermüller, Nicolas Kaeffer, Walter Leitner

**Affiliations:** † Max Planck Institute for Chemical Energy Conversion, Stiftstrasse 34-36, 45470 Mülheim an der Ruhr, Germany; ‡ Institut für Technische und Makromolekulare Chemie, RWTH Aachen University, Worringerweg 2, 52074 Aachen, Germany

## Abstract

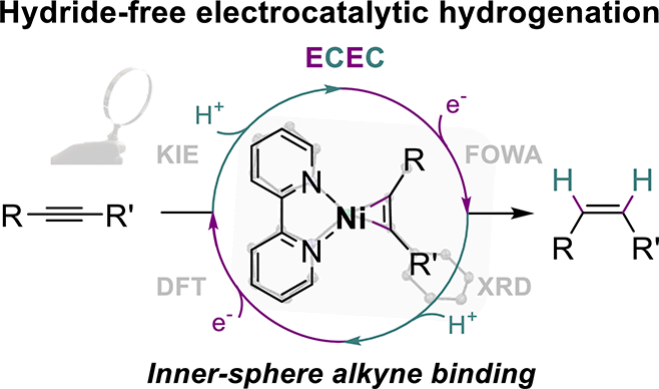

Hydrogenation reactions of carbon–carbon unsaturated bonds are central in synthetic chemistry. Efficient catalysis of these reactions classically recourses to heterogeneous or homogeneous transition-metal species. Whether thermal or electrochemical, C–C multiple bond catalytic hydrogenations commonly involve metal hydrides as key intermediates. Here, we report that the electrocatalytic alkyne semihydrogenation by molecular Ni bipyridine complexes proceeds without the mediation of a hydride intermediate. Through a combined experimental and theoretical investigation, we disclose a mechanism that primarily involves a nickelacyclopropene resting state upon alkyne binding to a low-valent Ni(0) species. A following sequence of protonation and electron transfer steps *via* Ni(II) and Ni(I) vinyl intermediates then leads to olefin release in an overall ECEC-type pattern as the most favored pathway. Our results also evidence that pathways involving hydride intermediates are strongly disfavored, which in turn promotes high semihydrogenation selectivity by avoiding competing hydrogen evolution. While bypassing catalytically competent hydrides, this type of mechanism still retains inner-metal-sphere characteristics with the formation of organometallic intermediates, often essential to control regio- or stereoselectivity. We think that this approach to electrocatalytic reductions of unsaturated organic groups can open new paradigms for hydrogenation or hydroelementation reactions.

## Introduction

The electrification of chemical processes is a major challenge to be met in the transition from petrochemical to defossilized production.^1−4^ Redox reactions are extensively present in chemical synthesis and provide a particularly relevant entry for electrons produced from renewables into the chemical value chains.^5−7^ In the specific case of hydrogenation reactions, which are conceptually simple but widely applied in bulk chemical processing as in fine synthesis,^8−10^ electrons can be directly used as reducing agents in combination with protons. Developing electrosynthetic strategies for the efficient and selective hydrogenation of organic unsaturated compounds would thus represent a major step forward. The electrochemical nature of these reactions may also lead to innovative reactivity patterns, which can be controlled or triggered using adequate electrocatalysts. In that aim, the tunability offered by transition-metal complexes place these species as ideal candidates in the exploration of the electrocatalytic space.

**Scheme 1:**
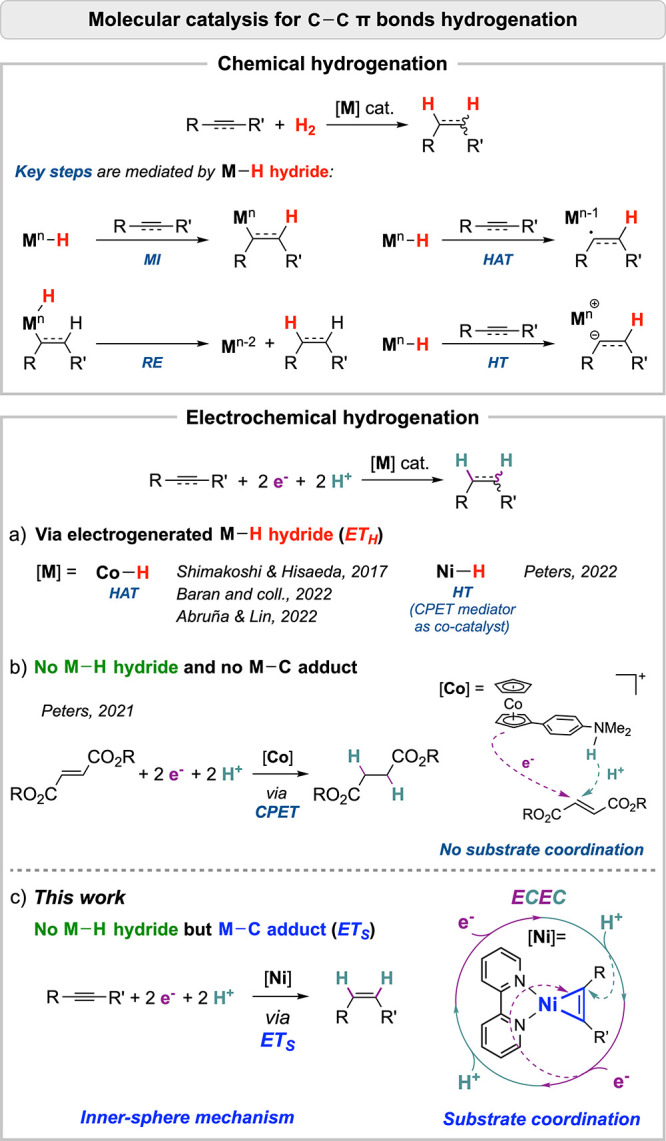
Main Mechanistic Routes for the Molecular (Electro)catalytic Hydrogenation of C–C Multiple Bonds

Most of the hydrogenation reactions of C–C unsaturated bonds are assumed or even proven to involve hydrides as catalytically relevant species.^10−15^ In the organometallic formalism, the reaction of a metal hydride with an unsaturated fragment can occur during four elementary key steps: migratory insertion (MI), hydride transfer (HT), hydrogen atom transfer (HAT), and reductive elimination (RE) (Scheme 1).^16,17^ Interestingly, these intermediates and steps are observed regardless whether the hydrogen source is molecular H_2_, hydride reagents, or protons in conjunction with electrons obtained from sacrificial reductants or an electrode. In the prototypical hydrogenations using H_2_ gas, metal hydrides are classically encountered both in the MI and the RE steps. Recent reports of C–C bond hydrogenations by transition-metal-based photocatalytic systems^18−20^ also propose metal hydride intermediates, except for one study suggesting a mechanism exempt of hydride.^19^

Molecular electrocatalysis has also been recently disclosed to achieve C–C unsaturated bond hydrogenation (Scheme 1), with metal hydrides highlighted as key intermediates.^21−23^ This point stands, for instance, in the electrochemical hydrogenation/deuteration of alkenes and alkynes using catalytic systems relying on a vitamin B_12_ model complex,^24^ a cobalt bipyridine complex,^25^ or salen complexes^25,26^ or the combination of a proton-coupled electron transfer (PCET) mediator with a nickel bis-diphosphine catalyst (Scheme 1a).^27^ These examples all share as common feature the generation of a M–H intermediate that further reacts with the C–C bond by hydride or hydrogen atom transfer.^24−27^ In such steps, electrons and protons are delivered together as H^–^ or H^•^, subscribing to pathways coined as electron transfer through hydride (ET_H_).^28^ At variance, the electrochemical hydrogenation of α,β-unsaturated esters directly catalyzed by a concerted proton–electron transfer (CPET) mediator (Scheme 1b) reported by Peters and co-workers^29^ is, to our knowledge, the only supported example of molecularly electrocatalyzed C–C hydrogenation where no hydride is involved. By ensuring a separate delivery of electron and proton, that strategy bypasses the ET_H_ route and thus doing outcompetes the undesired hydrogen evolution reaction (HER). In that case, however, the molecular construct of the CPET mediator precludes the formation of a metal–substrate adduct, often associated with improved selectivity and concertedness.^30,31^

Within that frame, our group has recently disclosed that [Ni(bpy)_3_]^2+^ is an efficient electrocatalyst for alkyne semihydrogenation providing the corresponding (*Z*)-olefins in good to high yields and faradaic efficiencies (FE).^32^ In the present work, we document that our system operates by an original pathway exempt of hydride intermediates. This mechanism instead involves a sequence of proton transfer (PT) and electron transfer (ET) steps from a nickelacyclopropene species as a resting state, as supported by organometallic, electrochemical, and kinetic studies (including isotope effect) as well as density functional theory (DFT) calculations. A most salient feature is that, while excluding the recourse to a catalytically competent hydride, the mechanism does proceed in the inner sphere of the metal *via* Ni-alkyne adduct formation and further PTs and ETs to the bound substrate. The system thus subscribes to the original frame of an ET_S_ mechanism (electron transfer to the substrate)^28^ in which electrons are delivered *via* the metal–substrate adduct and that remained so far elusive for electrocatalytic C–C hydrogenation (Scheme 1c). In addition, our findings also rationalize that the observed (*Z*)-stereoselectivity most likely ensues from a barrierless isomerization of two nickel vinyl isomers.

## Results and Discussion

### Initiation from [Ni(bpy)_3_]^2+^

**Scheme 2:**
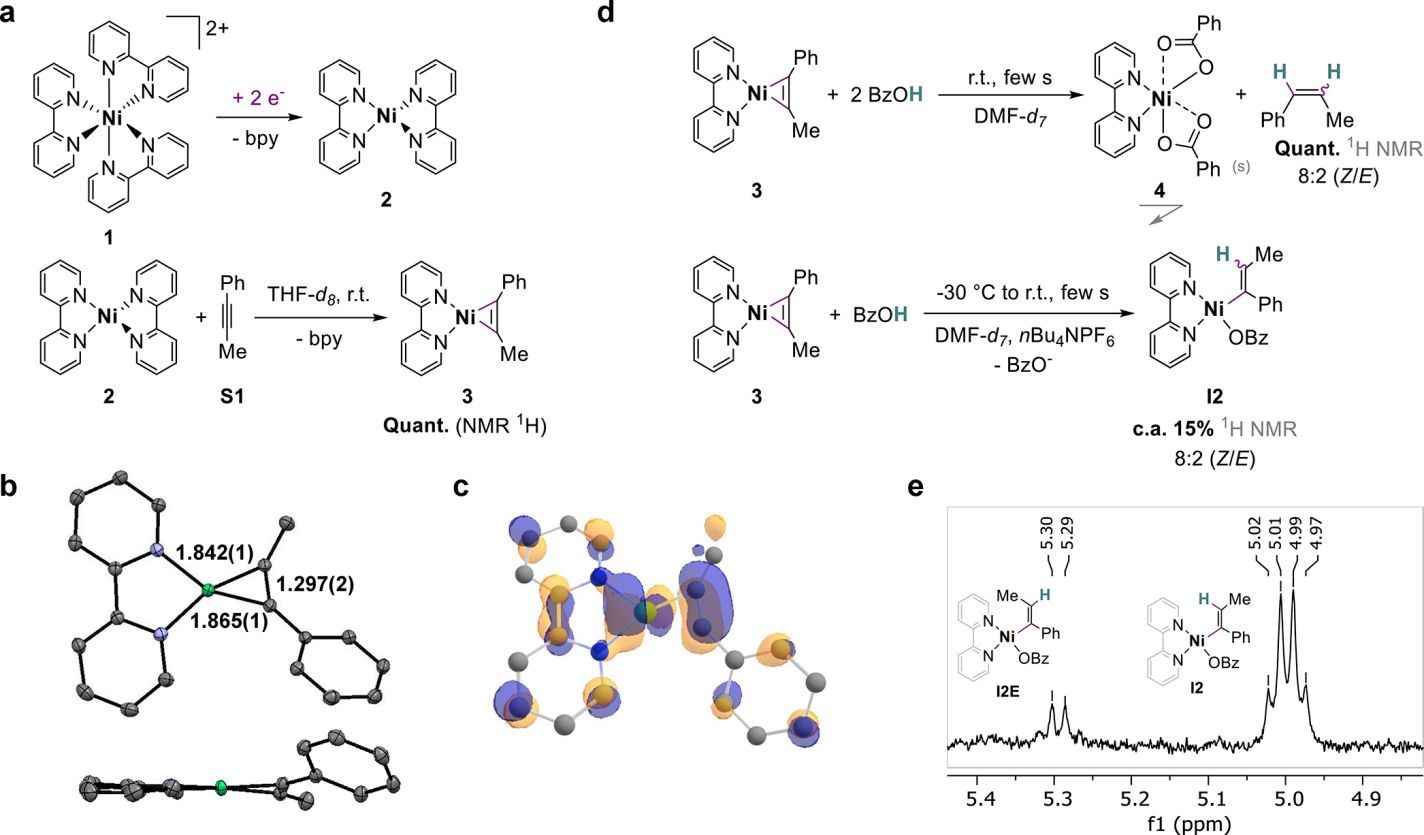
Generation, Structure and Protonation of Nickelacyclopropene **3** (a) Steps Involved in the Initiation Pathway and in the Synthesis of the Nickelacyclopropene **3**; (b) Molecular Structure of **3** Obtained by XRD (Front and Side Views; ORTEP; 50% Probability; H Atoms Omitted for Clarity; Color Code: Gray: C; Purple: N; Green: Ni); (c) Computed Electron Density of the HOMO of **3** (H Atoms Are Omitted for Clarity); (d) Protonation Reactions Starting from the Nickelacyclopropene **3**; and (e) ^1^H NMR Spectrum of the Vinyl Region of the Crude Mixture of **3** and BzOH (1 equiv) in DMF-*d*_7_ with *n*Bu_4_NPF_6_ (0.1 M).

Previous work of our group established that the complex [Ni(bpy)_3_](BF_4_)_2_ (**1**;2 BF_4_^–^) is a selective electrocatalyst for alkynes semihydrogenation into the corresponding (*Z*)-olefins.^32^ Electrochemical studies showed that the two-electron reduction of **1** is coupled with the release of a bpy (bpy = 2,2′-bipyridine) ligand evolving [Ni(bpy)_2_] (**2**) (Scheme 2a).^33^ Subsequent coordination of an alkyne at **2** was then suggested on the basis of cyclic voltammetry (CV) analysis and postulated to lead to a [Ni(bpy)(alkyne)] species upon displacement of a second bpy.^32,34^

To confirm the hypothesis and the nature of the generated complex, synthetically isolated **2** (obtained from [Ni(COD)_2_]; COD = 1,5-cyclooctadiene; see SI Section 3.1) was reacted with the model alkyne 1-phenyl-1-propyne (**S1**), upon inspiration from the literature.^35−38^ Addition of 1 equiv of **S1** to **2** in THF-*d*_8_ at room temperature (Scheme 2a) shows the formation of a dark red species. The spectroscopic signatures and molecular structure obtained by single-crystal X-ray diffraction (XRD) (Scheme 2b) identify the resulting isolated compound as the heteroleptic nickelacyclopropene complex [Ni(bpy)(PhCCMe)] (**3**), formed in an overall 65% yield from [Ni(COD)_2_] (see SI Section 3.2).^39^ In parallel of the synthetic isolation, the electrosynthetic formation of **3** was addressed. The passage of 2 electrons per **1** during bulk reductive electrolysis of a mixture of **1** and alkyne **S1** (**1**/**S1** 1:10 ratio, in 0.1 M *n*Bu_4_NPF_6_ DMF electrolyte) produces spectroscopic features revealing the formation of **3**, as in particular the two characteristic ^1^H NMR signals of the 6/6′ positions of the ligated bpy at 10.14 and 10.01 ppm (see SI Section 3.4.6). These results unambiguously demonstrate that the nickelacyclopropene complex **3** is evolved upon 2-electron reduction of **1** in the presence of the alkyne **S1**.

Having identified **3** as a plausible catalytically relevant species, we aimed at engaging the complex in electrocatalytic assays of alkyne semihydrogenation. However, we noted that the addition of benzoic acid (BzOH), a suitable proton source for our system,^32^ to the pre-electrolysis medium containing **3** readily results in the fading of the dark red solution. This observation suggests that **3** is converted in the presence of acid. This result was confirmed by an independent experiment, in which contacting 2 equiv of BzOH acid with **3** in THF-*d*_8_ produces a pale blue precipitate (Scheme 2d). ^1^H NMR analyses of the isolated solid dissolved in DMSO-*d*_6_ (low solubility) reveal signals with paramagnetic behavior and that can be attributed to [Ni(bpy)(BzO)_2_] (**4**) (see SI Sections 3.3 and 3.4.3). As a conclusion, **3** readily converts to **4** when BzOH is used as the proton source.

**Scheme 3:**
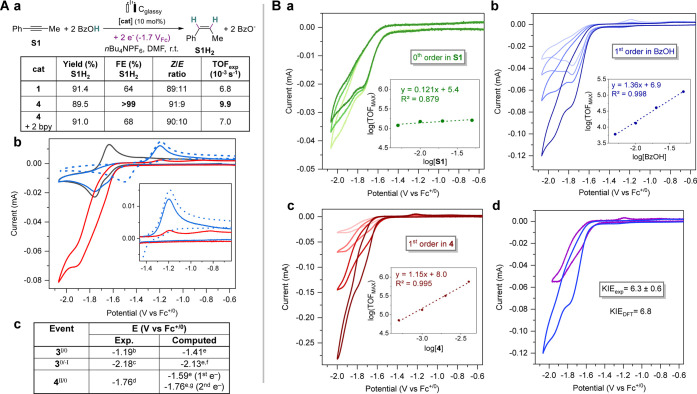
Electrochemical and Electrocatalytic Results (A) (a) Electrolysis with **1** (from ref (32)) or **4** as a Precatalyst. Yields, F.E., and *Z*/*E* Ratios Are Reported at Full Conversion; TOF_exp_ Values Are Estimated Based on Time to Full Conversion; (b) CVs of **3** Alone (Dotted Blue, Oxidation First), **4** Alone (Black), with **S1** (10 equiv) (Blue), or with **S1** and BzOH (50 equiv) (red); and (c) Experimental and Computed Redox Potentials (V vs Fc^+/0^) for Compounds **3** and **4**; (B) CVs of Mixtures of **4**, **S1**, and BzOH; (a) Increasing [**S1**] from 5 to 50 mM (Light to Dark Green) and Plot of log(TOF_MAX_) with Respect to log([**S1**]); (b) Increasing [BzOH] from 5 to 50 mM (Light to Dark Blue) and Plot of log(TOF_MAX_) with Respect to log([BzOH]); (c) Increasing [**4**] from 0.5 to 4 mM (Light to Dark Red) and Plot of log(TOF_MAX_) with Respect to log([**4**]); and (d) with BzOD (Purple) or BzOH (Blue); Conditions for Electrolysis: [Catalyst] = 1 mM, [**S1**] = 10 mM, [BzOH] = 100 mM, *E*_app_ = −1.72 ± 0.02 V_Fc_; Conditions for CV: Unless Otherwise Stated [**4**] = 1 mM, [**S1**] = 10 mM, [BzOH] = 50 mM, 0.1 V·s^–1^ as Scan Rate (ν); Supporting Electrolyte: DMF 0.1 M *n*Bu_4_NPF_6_. ^b^*E*_p,a_. ^c^*E*_1/2_. ^d^*E*_p,c_. ^e^*E*°. ^f^Quadruplet Configuration (See SI Section 5.2 for Other Spin Configurations). ^g^After the Release of One Benzoate Ligand (See Scheme S1).

We found that **4** is electrocatalytically active for the selective semihydrogenation of **S1** into (*Z*)-β-methylstyrene ((*Z*)-**S1H**_**2**_) and interestingly provides a large increase in faradaic efficiency (>99 vs 64%) and experimental turnover frequency (TOF_exp_; 9.9 vs 6.8 10^–3^ s^–1^) toward **S1H**_**2**_ compared to **1** (Scheme 3A(a)). In addition, when controlled potential electrolysis of **S1** using **4** is performed in the presence of excess bipyridine (2 equiv/**4**), TOF_exp_ (7.0 10^–3^ s^–1^) and FE (68%) decrease to levels consistent with that obtained using **1**. These results collectively suggest that excess free bipyridine in solution released upon reduction of [Ni(bpy)_3_]^2+^ or purposely added to **4** hinders the electrocatalytic semihydrogenation of interest. Thus, complex **1** was discarded in the rest of the study, and we instead focused on complexes **3** and **4** as these compounds derive from a more competent catalysis (see SI Section 2.2.2 for CV comparison of precatalysts **1** and **4**). We also note that the generation of heterogeneous deposits responsible for the electrocatalytic semihydrogenation could be discarded (see SI Section 2.3.2), in line with our previous report using **1**.^32^ With complexes **3** and **4** at hands, we further analyzed the framework of underlying electrochemical (E) and chemical (C) steps to withdraw mechanistic information.

### Initial Electron Transfer Steps

CV studies were conducted to understand the behavior of complex **4** under electrocatalytic conditions. The CV of **4** shows a pseudoreversible reduction characterized by a broad cathodic wave at *E*_p,c_ = −1.76 V vs Fc^+/0^ (abbreviated V_Fc_) featuring a shoulder at ca. −1.65 V_Fc_ and associated with a reoxidation wave at *E*_p,a_ = −1.64 V_Fc_ (Scheme 3A(b,c)).^40^ We tentatively attribute the broad cathodic wave of **4** to one-electron reduction events from Ni(II) to Ni(I) species and further to Ni(0) ones and that are closely located in potentials and underpinned by BzO^–^ ligand dissociation/association equilibria (see SI Section 2.2.4 for more details). Adding 10 equiv of the alkyne **S1** (Scheme 3A(b)) positively shifts both the shoulder of the reduction wave of **4** and in a drastic manner the reoxidation wave by 440 mV up to *E*_p,a_ = −1.20 V_Fc_. This latter value matches the oxidation potential of the native nickelacyclopropene **3** (*E*_p,a_ = −1.19 V_Fc_), which confirms the *in situ* formation of that species when **4** is doubly reduced in the presence of **S1**. A similar reoxidation event is observed when **1** is used instead (see SI Section 2.2.2). Adding BzOH to the previous mixture, a catalytic wave develops from the shoulder of the cathodic wave of **4** and reaches a first pseudoplateau at ca. −1.78 V_Fc_ and a second one at ca. −1.93 V_Fc_ (Schemes 3A(b) and Figure S6b). Interestingly, the reoxidation wave corresponding to the nickelacyclopropene species **3** can still be traced but is largely decreased (*i*_p,a_/*i*_p,a_^0^ = 0.13). Observing the nickelacyclopropene **3** under conditions of electrocatalytic turnover suggest that this species is the resting state in our conditions. Therefore, we posited that a protonation from **3** would be rate-determining. Pathways involving a stepwise electron transfer (ET) to **3** can be discarded under our electrocatalytic conditions since the applied potential is strongly positive to the reduction of **3** (*E*_app_ = −1.7 V_Fc_ vs *E*_1/2_(**3**^0/–I^) = −2.18 V_Fc_). We thus turned to the more detailed study of pathways where a proton transfer (PT) proceeds from **3**.

### Protonation Steps

The protonolysis of **3** with 2 equiv of BzOH in DMF-*d*_7_ at room temperature quantitatively evolves, along with the precipitation of **4** (*vide supra*), the olefinic hydrogenation product **S1H**_**2**_ in a *Z*/*E* ratio of 8:2 (see SI Section 3.4.3). This observation clearly indicates that a two-fold protonation is accessible at **3**. The same experiment in the presence of the supporting electrolyte *n*Bu_4_NPF_6_ (0.1 M) induces solubilization of the paramagnetic species **4** which prevents a sensible ^1^H NMR analysis. To tentatively trap the protonation sequence after the first PT, we contacted **3** with only 1 equiv of BzOH and at low temperature (−30 °C) in DMF-*d*_7_ with 0.1 M *n*Bu_4_NPF_6_ (Scheme 2d). The ^1^H NMR spectrum of the reaction mixture shows the appearance of two quadruplets at 5.00 and 5.30 ppm, which we attribute to α-methyl vinylic protons of putative nickel(II) vinyl species (**I2** and **I2E**, Scheme 2e), based on the literature^41^ and the shifts expected for the corresponding free olefins. We attribute the build-up of two vinylic signals to the presence of the (*Z*)- and the (*E*)-vinyl complexes, respectively (see SI Section 3.4.2), found in a *Z*/*E* 8:2 ratio very close to the one of alkenes generated under stoichiometric (using 2 equiv of BzOH) and electrocatalytic conditions. The evolution of a Ni-vinyl species was also observed when using diphenylacetylene as the alkyne substrate (see SI Section 3.4.5). The facile protonation at the nickelacyclic carbons in **3** is further rationalized by the strong electron density located at these carbons in the highest occupied molecular orbital (HOMO) of this complex (Scheme 2c). However, only the vinyl protons in α-methyl position could be observed, which strongly support that the protonation is regioselective for that position. In the absence of *n*Bu_4_NPF_6_, the vinylic signals were not obtained and the benzoic acid (1 equiv) was fully consumed toward the formation of alkenes. We surmise that, without *n*Bu_4_NPF_6_, precipitation of **4** constitutes a driving force toward the formation of alkenes preventing the observation of the nickel vinyl intermediates. We also found that a similar body of results is obtained when **3** is electrogenerated from **1**, followed by addition of BzOH in the mixture (see SI Section 3.4.6).

The observation of a Ni(II) vinyl intermediate indicates that the lifetime of such species may be long enough to afford for ET, in the case of electrocatalytic turnover. As such, this vinyl intermediate can constitute a bifurcating point between two mechanisms, namely, an EECC-type one where the two PTs are successive and an ECEC-type one, where the second PT is preceded by ET.^42^

### Electrocatalytic Cycle

**Scheme 4:**
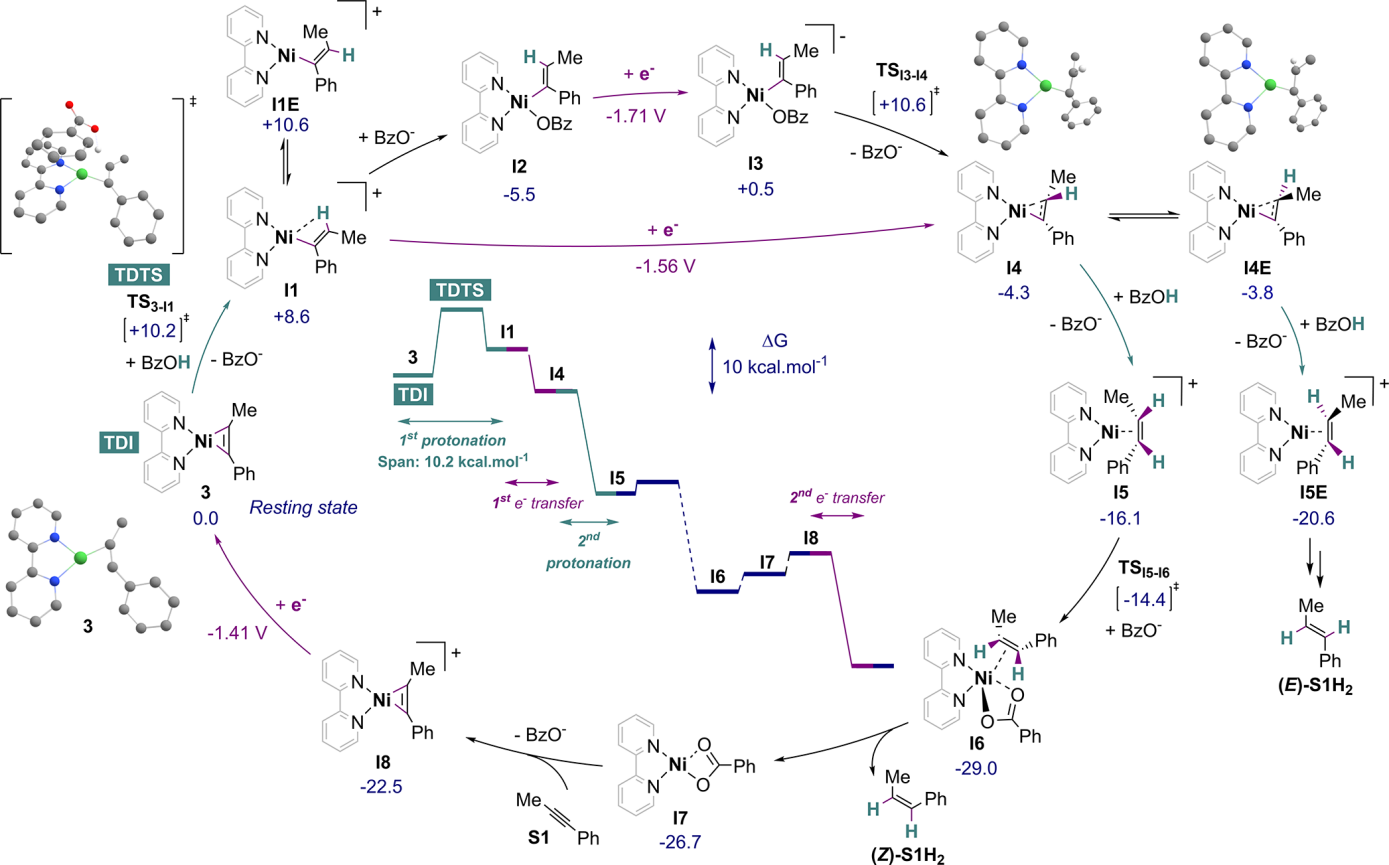
Computed ECEC-Type Mechanism of the Electrocatalytic Semihydrogenation of Alkyne **S1** with **3** Δ*G* values are given in kcal mol^–1^ and reaction coordinates are depicted in the middle of the cycle. Conditions: PBE-D3/6-311+G(d,p) level of theory and CPCM model to account for solvent effect (DMF). Geometries of **3**, **TS_3-I1_**, **I4**, and **I4E** are given with H atoms omitted for clarity (except vinylic).

To gain further information on the mechanism, kinetic data were extracted from CV experiments. As we could not identify suitable conditions leading to a canonical S-shaped CV, we resorted to foot-of-wave analysis (FOWA).^43^ Regardless of the mechanism considered (EECC or ECEC), FOWA can be used to provide an estimate of the maximal turnover frequency (TOF_MAX_; see SI Section 4 for details).^43^ Following this methodology, we have found an average TOF_MAX_ of 1.37 ± 0.09 10^5^ s^–1^, leading to a reaction span of 10.5 ± 0.4 kcal mol^–1^ (see SI Section 4). Applying the same methodology while varying the concentrations in reaction partners, we obtained that the reaction is first order in the catalyst **4**, zeroth order in alkyne, and first order in BzOH (Scheme 3B). Moreover, a kinetic isotope effect (KIE) of 6.3 ± 0.6 derives from FOWA analyses of CVs recorded using either BzOH or BzOD. Taken together, these results support that a protonation of a nickel-based intermediate after alkyne binding is rate-determining. Capitalizing on the kinetic data and reactivities highlighted in the previous sections, we discuss now possible mechanistic pathways by putting DFT calculations (Scheme 4) in perspective with experimental results.

Electrochemical studies support that **3** is the resting state in the catalytic cycle, which was thus taken as the entry point and as origin of our thermodynamic scale for calculations. While the reduction of **3** was ruled out (*vide supra*), protonolysis of this species observed above indicates that protonation is feasible at this stage. A first outer-sphere protonation of **3** by BzOH was indeed found accessible (Δ*G*^‡^(**TS**_**3-I1**_) = +10.2 kcal mol^–1^) and leads to a cationic Ni(II) vinyl species **I1** at +8.6 kcal mol^–1^, which undergoes barrierless coordination of a benzoate ligand to give **I2** (Δ*G* = −5.5 kcal mol^–1^). This thermodynamically favored protonation yielding **I2** is in good agreement with the NMR investigation (*vide supra*).

This first protonation then leads to a bifurcation point between the EECC- and ECEC-type pathways. In the case of EECC routes, the first protonation is followed by a second PT, before ET occurs. The most favorable second PT (outer sphere) was found to proceed *via* a TS at +15.6 kcal mol^–1^ (see SI Section 5.4.1). The span associated with this reaction path would then be 21.1 kcal mol^–1^, which is substantially higher than the experimental estimate (10.5 ± 0.4 kcal mol^–1^). Therefore, EECC-type pathways have been discarded (see SI Section 5.4.1 for details), and we thus turned to scrutinize in more detail possible pathways of the type ECEC.

In this case, the reductions of cationic or neutral vinyl species **I1** or **I2** are computed at *E*^0^ = −1.56 and −1.71 V_Fc_, respectively, which are more anodic than or close to our applied potential and thus possible under our conditions. Regardless of the relevant Ni(II) vinyl intermediate, subsequent ET is computed to lead in turn to a nickel(I) vinyl species **I4** (Δ*G* = −4.3 kcal mol^–1^). From **I4**, the different transition states obtained for inner-sphere protonation with BzOH coordinated were all found to be very high in energy (>24 kcal mol^–1^, see SI Section 5.4.3, Scheme S5).

In contrast, outer-sphere protonation by BzOH is barrierless and thus the most plausible next step, giving the nickel alkene complex **I5** at −16.1 kcal mol^–1^. At this stage, coordination of a benzoate ligand is easy *via***TS**_**I5-I6**_ at −14.4 kcal mol^–1^ and readily triggers the release of the alkene **S1H**_**2**_ from the resulting alkene benzoate adduct intermediate **I6** (Δ*G* = −29.0 kcal mol^–1^). The decoordination of the alkene leads to the Ni(I) benzoate complex **I7** at −26.7 kcal mol^–1^. The favored binding of alkyne **S1** to **I7** displaces the BzO^–^ ligand and generates **I8**, the cationic analogue of **3** (Δ*G* = −22.5 kcal mol^–1^). Electron transfer on this intermediate (*E*^0^ = −1.41 V_Fc_) yields **3** and thus closes the catalytic cycle. A pathway including the reduction of **I7** prior to alkyne coordination is also accessible, although at a slightly more negative potential (*E*^0^ computed at −1.76 V_Fc_) and is further discussed in SI Section 2.2.5.

In the ECEC frame, whether **I1** undergoes BzO^–^ coordination to **I2** or a direct reduction to **I4** is arguable. The first pathway defines an energetic span scaling to 16.1 kcal mol^–1^ between **I2** as a TOF-determining intermediate (TDI) and BzO^–^ decoordination **TS**_**I3-I4**_ as TOF-determining TS (TDTS). The second one displays a span of 10.2 kcal mol^–1^ between **3** and **TS**_**3-I1**_, which corresponds to the first PT. In the first case yet, a dependence of the apparent reaction rate on [H^+^] is not to be expected, contradicting experimental findings (*vide supra*). For that reason and because of the lower span offered by the second pathway, we favor the hypothesis of the direct reduction of **I1** to **I4**, with rate-determining protonation of **3**.

Considering this pathway, the computed ECEC-type mechanism agrees with kinetic results obtained from experiments. In particular, the calculated span (10.2 kcal mol^–1^) is in good match with the expected span of 10.5 ± 0.4 kcal mol^–1^ derived from experimental TOF_max_. In addition, the rate-limiting first protonation of **3** is corroborated by experimental orders in reaction partners (*vide supra*) and good agreement is found between experimentally observed and computed KIEs for that first protonation (6.3 ± 0.6 vs 6.8, respectively; see SI Section 5.3). An alternative and close pathway involving a concerted proton–electron transfer from **3** to **I4** could also be considered. The BDE of **I4** was calculated to be 65.2 kcal mol^–1^. This value is thermodynamically suitable for such a pathway.^44,45^ The latter can therefore not be fully excluded compared to the stepwise PT-ET sequence from **3**.

We stress that the ECEC-type mechanism proposed was identified as the most favored one under our conditions of the applied potential (*E*_app_ ≈ −1.7 V_Fc_), although we recognize that initiation processes and evolution of BzO^–^ may limit the direct quantitative comparison between experimental and computed kinetic values. Especially, the build-up of BzO^–^ concentration at advanced alkyne conversion is likely to shift the mechanism into a pathway operating at a slightly more negative potential, *via* the reduction of **I7** preceding alkyne binding into **3** and that also gives good agreement between electrochemical and computational results. At more negative applied potentials (*E*_app_ ≤ −1.8 V_Fc_), we note a strong degradation of F.E. (Figure S8), which is suggestive that other, less selective mechanistic pathways come into play. These points are further discussed in SI Section 2.2.5.

It is interesting to mention here that the ECEC-type mechanism formally consists in the hydrogenation of a ligand—in that case, an alkyne—which is well-known as a degradation pathway of molecular electrocatalysts^46−55^ or sometimes as an initiation to access more active catalytic species.^56−58^ In the present work, this phenomenon is desired and fully exploited as it is part of the catalytic cycle. The absence of substantial decomposition may be here due to the stabilization of the nickel catalyst by benzoate ligands. We also note that the protonation of metallacyclopropenes into vinyl intermediates and further leading to olefin release is not an unprecedented reactivity. Such a pattern was, for instance, explored with early transition-metal complexes, typically [M(Cp)_2_(alkyne)] metallocenes with M = Ti, Zr, Hf.^59−62^ However, these systems have, to our knowledge, not been exploited under catalytic conditions. The generation of oxygenated bases upon protonation of the metallacyclopropene by R–OH acids usually results in the formation of strong M–O bonds that irreversibly poison the catalyst and thus preclude turnover. In our case, we surmise that the comparatively lower oxophilicity of Ni combined with the electroreductive conditions enable to displace the bound benzoate ligand and thus entry into a catalytic cycle.

### Stereoselectivity

On the basis of the identified ECEC-type mechanism, we then addressed the experimentally observed (*Z*)-selectivity (see SI Section 5.4.3 for the different isomerization pathways investigated). We first considered the isomerization of the Ni(II) vinyl complexes **I1** or **I2**. The isomerization of the first intermediate **I1** is possible without a transition state and leads to **I1E** (Δ*G* = + 10.6 kcal mol^–1^). In contrast, for the neutral Ni(II) vinyl species **I2**, the TS associated with such a transformation was found to be very high in energy (>30 kcal mol^–1^) which makes that transformation very unlikely. Despite this unreachable barrier at **I2**, the observation of the two isomers **I2** and **I2E** upon stoichiometric protonation of **3** (see Scheme 2d,e) can be rationalized in virtue of the easy (*Z*)–(*E*) isomerization at the preceding intermediate **I1**. Isomerization at the following Ni(I) vinyl species **I4** was also considered. The corresponding isomers, **I4** and **I4E**, are both accessible at −4.3 and −3.8 kcal mol^–1^, respectively (Scheme 4), and also interconvert *via* a barrierless isomerization. Isomerization at a later stage from a Ni–olefin complex would imply oxidative addition of a C–H olefinic bond into a hydrido-vinyl species, which we rule out (*vide infra*), or a hydrogenation/dehydrogenation sequence that does not agree with the absence of overhydrogenation products. (*Z*)–(*E*) isomerizations at **I1** or **I4** intermediates are thus the most plausible options under electrocatalytic conditions and the predominance of one versus the other can hardly be assessed. We yet note that **I4** follows **I1** and that the Gibbs free energy difference for **I4** and **I4E** of 0.5 kcal mol^–1^ is relatively close to the difference of 1.3 kcal mol^–1^ expected for a Boltzmann distribution of 9:1 (*Z*/*E*), which is the experimental selectivity for the alkene products after electrolysis (*vide supra*). If stereoselectivity is determined at **I4**, protonolysis of the nickel–carbon bond by benzoic acid approaching **I4** or **I4E** would lead to the (*Z*)- or the (*E*)-olefin, respectively, and in turn generate the *Z*/*E*-partitioned product mixture.

### Hydride Pathway

**Scheme 5:**
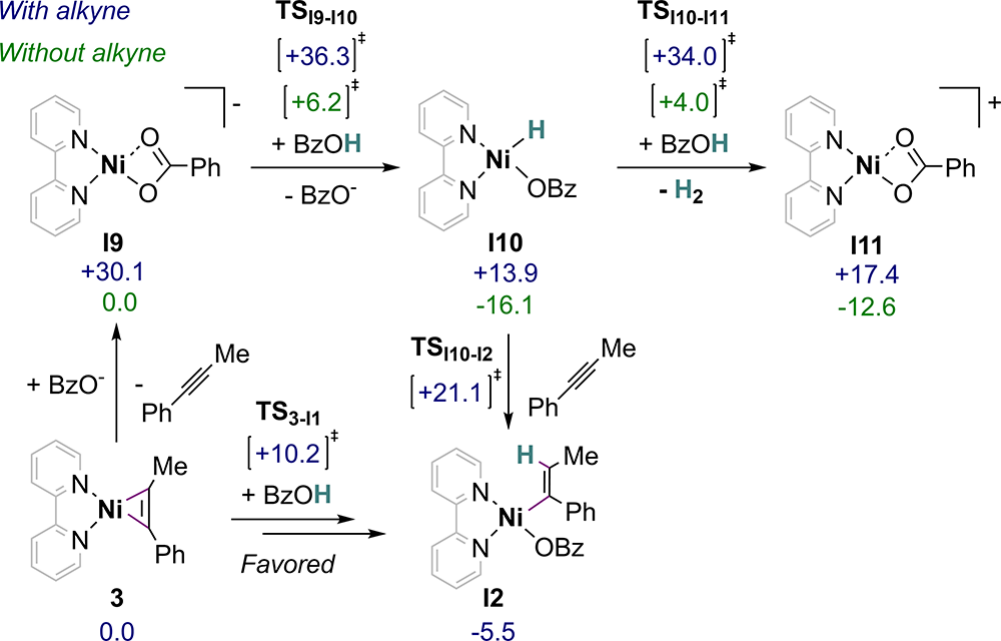
Key DFT Calculation Findings on Nickel Hydride Formation and Reactivity ΔG values are given in kcal mol^–1^. Conditions: PBE-D3/6-311+G(d,p) level of theory and CPCM model to account for solvent effect (DMF).

While the most plausible mechanistic route identified so far does not involve a hydride, we wanted to evaluate the possible role of such a common intermediate in hydrogenation, participating to migratory insertion, hydride or hydrogen atom transfer, or reductive elimination (*vide supra*, Scheme 1).^11,12,17^ Among hydride species conceivable in our system, several could be discarded due to exceedingly endergonic formation (see SI Section 5.4.2), leading to consider a Ni(II) hydride [Ni(H)(bpy)(BzO)] (**I10**) as the most plausible candidate (Scheme 5).

Despite an endergonic formation (Δ*G* = +13.9 kcal mol^–1^), the hydride species **I10** is accessible at room temperature and would proceed by protonation of the anionic Ni(0) benzoate **I9** intermediate with BzOH. However, two facts speak against such a pathway. Not only the protonation of **I9** displays a high energetic barrier (Δ*G*^‡^(**TS**_**I9-I10**_) = +36.3 kcal mol^–1^) but also, in the presence of an alkyne, the formation of **I9** itself is strongly disfavored (Δ*G* = +30.1 kcal mol^–1^) due to the high stability of the resting state **3**. These results suggest that the presence of the alkyne, and therefore **3**, prevents the formation of a hydride and thus hydrogen evolution by protonation of the latter. Such points are well in line with the electrocatalytic selectivity of **4** toward alkyne semihydrogenation, as evidenced by a quantitative faradaic efficiency to the olefin and the absence of detectable amounts of hydrogen in the headspace (see SI Section 2.1.3). We note, however, that, in the absence of the alkyne, a catalytic pathway for the hydrogen evolution can be located by computation. The associated span of 20.1 kcal mol^–1^ is consistent with a reaction at room temperature. These computational findings are in agreement with the evolution of H_2_, although poorly effective (turnover number: TON = 0.9, TOF_exp_ = 0.19 10^–3^ s^–1^, FE(H_2_) = 27%), observed under electrocatalytic conditions in the absence of the alkyne (see SI Section 2.2.3 for CV of **4** in the presence of BzOH only).

## Conclusions

The electrochemical semihydrogenation of alkynes catalyzed by homogeneous bipyridine-based nickel complexes [Ni(bpy)*_n_*(X)*_m_*]^q^ has been recently achieved. We have shown here by a combined experimental and theoretical study that, under our conditions, a mechanism of the type ECEC is the most favorable pathway for that reaction. In particular, we found that a reductively induced nickelacyclopropene intermediate is the resting state, from which protonation proceeds as the (genuine) rate-determining step of the catalytic cycle. The selectivity of the reaction was rationalized to be governed by the formation of vinylic intermediate isomers close in energy, at either Ni(II) or Ni(I) stages. Most importantly, our results strongly support that no metal hydride species is involved in the catalytic activity of semihydrogenation in our conditions. We found that the absence of a competent hydride intermediate ensues from the presence and coordination of the alkyne substrate (ET_S_ pathway). The bypass of a hydride is actually possible due to a sequence of electron and proton transfers directly at the Ni-coordinated unsaturated fragment. In addition, triggering a pathway exempt of hydride intermediates also eliminates the hydrogen evolution reaction as a side-reaction. While established systems for the catalytic hydrogenation of C–C unsaturations are so far proven to involve hydride species in migratory insertion, reductive elimination, hydride or hydrogen atom transfer steps (ET_H_), we document here an alternative ET_S_ mechanism exempt of such hydride intermediates, but that maintains catalysis at the metal center. Such inner-sphere character is a feature of organometallic catalysis that is often integral to the control of regio- or stereoselectivity and brought here to electrocatalytic hydrogenation of C–C multiple bonds. We believe that this original approach to the reduction of organic unsaturations will open new paradigms for hydrogenation or hydroelementation reactions, for instance, with systems able to decouple proton and electron deliveries such as redox, photocatalytic, or electrocatalytic approaches.

## Supporting Information Available

Detailed descriptions of experimental methods, additional electrochemical and electrocatalytic data (e.g., CVs of **1**, **3**, **4**, electrolyses at different applied potentials), additional mechanistic details (e.g., [BzO^–^] effects), synthesis of compounds and stoichiometric experiments with associated characterizations (e.g., ^1^H, ^13^C NMR), and additional computational data (e.g., computed Gibbs energies for an EECC mechanism) (PDF)
