# Supplementary material for: Hydride-Free Hydrogenation: Unraveling the Mechanism of Electrocatalytic Alkyne Semihydrogenation by Nickel–Bipyridine Complexes
Source: J Am Chem Soc. 2023 Jul 25;145(31):17103–11. doi: 10.1021/jacs.3c03340 (PMC10416305; doi:10.1021/jacs.3c03340)
Supplement: Supplementary file 1 — ja3c03340_si_001.pdf [file ja3c03340_si_001.pdf]

Supporting Information

Hydride-free Hydrogenation: Unraveling the Mechanism of Electrocatalytic Alkyne Semihydrogenation by Nickel–Bipyridine Complexes

Gabriel Durin,[a] Mi-Young Lee,[a] Martina A. Pogany,[a] Thomas Weyhermüller,[a] Nicolas Kaeffer,[a]* Walter Leitner [a,b]

[a]*Max Planck Institute for Chemical Energy Conversion, Stiftstrasse 34-36, 45470 Mülheim an der Ruhr, Germany*
[b]*Institut für Technische und Makromolekulare Chemie, RWTH Aachen University, Worringerweg 2, 52074 Aachen, Germany*

*Corresponding author: Nicolas Kaeffer (nicolas.kaeffer@cec.mpg.de)

1. General consideration **4**

2. Electrochemical experiments **5**

2.1. Procedures 5

2.1.1. Cyclic voltammetry *5*

2.1.2. Electrolysis *5*

2.1.3. Analytical methods *6*

2.2. Additional electrochemical data 7

*2.2.1. Redox couples of **3*** *7*

*2.2.2. Comparison of redox behaviour of **1** and **4*** *7*

*2.2.3. Behaviour of **4** in presence of benzoic acid* *8*

*2.2.4. Behaviour of **4** in presence of benzoate* *8*

2.2.5. Additional mechanistic details *11*

2.3. Additional electrolysis results 13

2.3.1. Applied potential dependence *13*

2.3.2. Assessment of electrocatalytically active deposits *13*

3. Spectroscopic characterizations **16**

3.1. Synthesis of [Ni(bpy)$_2$] (**2**) 16

3.2. Synthesis of [Ni(bpy)(PhCCMe)] (**3**) 18

3.3. Synthesis of [Ni(bpy)(BzO)$_2$] (**4**) 20

3.4. Stoichiometric experiments 21

*3.4.1. [Ni(bpy)$_2$] (**2**) + 1-ph-1-propyne (**S1**)* *21*

*3.4.2. [Ni(bpy)(PhCCMe)] (**3**) + benzoic acid (BzOH)* *22*

*3.4.3. [Ni(bpy)(PhCCMe)] (**3**) + 2 BzOH* *23*

*3.4.4. [Ni(bpy)$_2$] (**2**) + BzOH* *24*

*3.4.5. [Ni(bpy)(PhCCPh)] (**5**) + BzOH* *25*

3.4.6. Electrochemical stoichiometric experiments *26*

3.5. XRD section 28

4. Foot-of-the-wave (FOWA) analysis **29**

5. Computational details **31**

5.1. General consideration 31

5.2. Spin configurations 31

5.3. Computed KIE 31

5.4. Additional computational results 31

5.4.1. Computed EECC pathway *31*

5.4.2. Hydride pathways 33
5.4.3. Stereoisomers, regioisomers and alternative transition states 33
5.5. Three lowest frequencies and Gibbs free energy for all computed structures 35

6. References 48

1. General consideration

Synthetic manipulations were performed under inert conditions of argon atmosphere either in an MBRAUN UNILAB Plus glove box or using standard Schlenk techniques, in oven-dried glassware. Organic solvents (analytical grade; Carl Roth) other than DMF were dried and degassed by passage over an MBRAUN SPS-7 solvent purification system, handled under argon atmosphere and stored over molecular sieves. 2,2-Bipyridine (≥99%) was purchased from Alfa Aesar (Germany) and *N,N*-Dimethylformamide (DMF, 99.8%, Extra Dry over Molecular Sieve) was obtained from ACROS Organics. Tetrabutylammonium hexafluorophosphate (nBu$_4$NPF$_6$) (for electrochemical analysis, ≥ 99.0%), tetrabutylammonium benzoate (nBu$_4$NBzO) (for electrochemical analysis, ≥ 99.0%), 1-phenyl-1-propyne (**S1**) (99%), and benzoic acid (BzOH) (≥99.5%) were all purchased from Merck (Darmstadt, Germany). [Ni(COD)$_2$] (98%) was obtained from ABCR GmbH. Deuterated benzoic acid (BzOD) was obtained from Chem Cruz™. The chemicals in the solid state were dried under vacuum at 110 ºC over 6 hours. NMR solvents were degassed by three freeze-pump-thaw cycles and dried over molecular sieves. [Ni(bpy)$_3$](BF$_4$)$_2$ (**1**;2 BF_4^-)[1] and [Ni(bpy)(PhCCPh)] (**5**)[2] were prepared according to literature procedures.

NMR spectra were recorded on a Bruker AVANCE NEO 400 MHz NMR spectrometer with a Bruker Prodigy probe at the indicated temperature. Chemical shifts (δ) are given in parts per million related to tetramethylsilane (TMS) and the coupling constants (J) in hertz. The solvent residual signal was used as a reference. First-order spin multiplicities are abbreviated as singlet (s), doublet (d), triplet (t), and quadruplet (q). Couplings of higher-order or overlapped signals are denoted as m (multiplet).

Elemental analysis (C, H, and N) was performed on an Elementar UNICUBE fitted with a thermal conductivity detector. Single crystals of compounds CCDC 2226437 were selected under a microscope in polarized light with an applied nitrogen cryostream at approximately −40 °C and covered with polyfluorinated polyether. The crystals were picked up with nylon loops and rapidly mounted in the nitrogen cold gas stream of the diffractometer at 100 K to prevent solvent loss. A Bruker D8 Venture diffractometer equipped with a IµS3 Diamond source, INCOATEC Helios mirror optics (Mo Kα radiation; λ = 0.71073 Å), and a Photon III detector were used for data collection. Data were processed using the Bruker APEX 3 software suite. The final cell constants are based on refinement of the XYZ centroids of several thousand reflections above 20 σ(I). Structures were solved and refined using the embedded Bruker SHELXTL software package. All non-H atoms were anisotropically refined, and H atoms were placed at calculated positions and refined as riding atoms with isotropic displacement parameters.

2. Electrochemical experiments

2.1. Procedures

All electrochemical experiments were performed in DMF 0.1 M nBu$_4$NPF$_6$ electrolyte solution at room temperature with a catalyst concentration (when present) of 1 mM. A AgCl/Ag (leak-free type, OD of 5 mm, Innovative Instruments, Inc.) was used as reference electrode unless otherwise specified. The electrolyte and analyte mixtures were introduced in the electrochemical cell under Ar flow.

2.1.1. Cyclic voltammetry

Cyclic voltammograms (CVs) were recorded with a VSP-300 (Biologic Science Instruments, France) potentiostat equipped with an analogic ramp generator module. A glassy carbon disk (3 mm diameter, ALS Co., Ltd, Japan) and a coiled platinum wire (0.5 mm diameter, ALS Co., Ltd, Japan) were used as working and counter electrodes, respectively. The one-compartment electrochemical glass cells were filled with 5 mL of the electrolyte solution, and purged with Ar at least for 10 min. The working electrode was polished over an alumina polishing pad using a polishing alumina (0.05 µm, ALS Co., Ltd) followed by rinsing with deionized water and ethanol. Reference and counter electrodes were rinsed with ethanol. Electrodes were dried under a stream of argon prior to insertion in the cell. Stock solutions (1 M) in electrolyte (DMF, 0.1 M nBu$_4$NPF$_6$) were used for the addition of reaction partners to the electrochemical cell. After each CV measurement, the working electrode was taken from the cell and freshly polished. Unless otherwise noted, the CVs were recorded at a scan rate (ν) of 0.1 V·s^{-1}. Ohmic drop compensation (85%) was applied. Reported potentials in CVs are referenced to the ferrocenium/ferrocene (Fc$^{+/0}$) couple (abbreviated to V$_{Fc}$) by adding ferrocene at the end of the measurements. In the case of **3**, CVs were conducted in glovebox.

2.1.2. Electrolysis

Electrolyses were carried out using a SP-300 (Biologic Science Instruments, France) potentiostat. Electrolyses were performed in a customized H-type glass cell, having anode and cathode chambers separated by the glass frit (P3 pore size). A carbon foam (0.6 cm × 0.6 cm × 2.4 cm; VC003825, Goodfellow Cambridge Ltd, UK) and platinum mesh (20 x 20 mm, 0.1 mm thickness, Goodfellow Cambridge Ltd, UK) were used as working and counter electrodes, respectively. The appropriate volume of electrolyte was introduced in each chamber of the electrochemical cell and sparged with Ar for 5 min. In a typical experiment, 5 µmol of **1** or **4** and 50 µL of a 1 M mesitylene (internal standard) solution in DMF 0.1 M nBu$_4$NPF$_6$ (final concentrations of **1** or **4** and mesitylene 10 mM) were then introduced in the cathode chamber, followed by the additions of electrolytes 1 M in the alkyne under consideration and 1 M in benzoic acid. The final volume of solution in each chamber was set to 5 mL.

Unless otherwise noted, the concentrations in catalyst, alkyne and benzoic acid are respectively [**1**] or [**4**] = 1 mM, [**S1**] = 10 mM, [BzOH] = 100 mM.

Right before electrolysis, the ohmic drop in the cell (R$_{cell}$) was determined. Then, electrolyses were performed under potentiostatic conditions, with no ohmic drop compensation applied. The reported electrolysis potentials are corrected *a posteriori* for ohmic drop (I.R$_{cell}$; 100%) and found at $E_{app} \approx -1.7$ V$_{Fc}$, unless otherwise noted.

The cathodic electrolyte was continuously purged with Ar (ca. 1 mL·min^{-1}) during the run of electrolysis. Aliquots of ca. 200 µL from the cathode chamber were collected during the electrolysis based on the time or charge passed and

analyzed by gas chromatography (GC) with the samples before and after the reaction. In addition, the cathodic chamber was sampled (150 µL aliquots) prior to and at the end of the electrolysis, the aliquots mixed with 250 µL deuterated chloroform and analyzed by ^{1}H nuclear magnetic resonance (NMR).

2.1.3. Analytical methods

Samples were analyzed by gas chromatography using a gas chromatograph equipped with a flame ionization detector (GC-FID; Nexis GC-2030, Shimadzu, Japan) with elution over a Rtx-1 column (30 m × 0.25 mm with 0.5 µm film thickness, Restek Corp., USA) using He as a carrier gas and a gas chromatograph equipped with a mass spectrometer (GC-MS; QP2020 NX, Shimadzu, Japan) with elution over a Rtx-1 column (30 m × 0.25 mm with 0.5 µm film thickness, Restek Corp., USA) using He as a carrier gas.

Integrals of the GC-FID peaks of the substrates and products were normalized over the one of the internal standard (mesitylene) for quantification. The quantification of carbon balance, alkyne conversion, alkenes yield, faradaic efficiency (FE) towards alkenes and turnover numbers (TONs) were calculated using the following equations:

$$\text{Carbon balance (\%)} = \frac{C_t(\mathbf{S}) + C_t(\mathbf{SH_2})}{C_i(\mathbf{S}) + C_i(\mathbf{SH_2})} \times 100 \qquad (\mathbf{1})$$

$$\text{Conversion (\%)} = \frac{C_i(\mathbf{S}) - C_t(\mathbf{S})}{C_i(\mathbf{S})} \times 100 \qquad (\mathbf{2})$$

$$\text{Selectivity} = \frac{C_t(\mathbf{SH_2})}{C_i(\mathbf{S})\text{-}C_t(\mathbf{S})} \qquad (\mathbf{3})$$

$$\text{Yield (\%)} = \text{conversion} \times \text{selectivity} \qquad (\mathbf{4})$$

$$\text{TON} = \frac{n_t(\mathbf{SH_2})}{n_i(\mathbf{Ni})} \qquad (\mathbf{5})$$

$$\text{FE (\%)} = \frac{2 \times n_t(\text{SH}_2) \times \text{F}}{Q_t} \times 100 \qquad (\mathbf{6})$$

Where $C_i(\mathbf{S})$, $C_t(\mathbf{S})$, $C_i(\mathbf{SH_2})$ and $C_t(\mathbf{SH_2})$ are concentrations in alkyne **S** or alkene **SH$_2$** at the beginning of reaction (C_i) and at the given time (C_t), $n_t(\mathbf{SH_2})$ is the amount of alkene at a given time, $n_i(\mathbf{Ni})$ is the amount of **Ni** at the beginning of the reaction, Q_t is the charge passed through the system at a given time and F is the Faraday constant (96485 C·mol^{-1}).

Headspace analyses were performed in a custom two-compartment H-type glass cell that differs from SI section 2.1.2 in size and tightness. The final solution volume was set to 20 mL (12.5 mL of which was in the cathode compartment). Electrolyses were performed as described in SI section 2.1.2. Gas samples were taken directly from the electrolysis cell by an AOC-6000 auto sampler and analyzed by a Shimadzu GC-2030 gas chromatography eluting over a Carboxen 1010 PLOT capillary column using N_2 as carrier gas and equipped with a TCD-detector. The lower limit of detection for H_2 in that configuration is 0.2%v (in volume) which corresponds to 5 µmol H_2 in the ca. 78 mL cell (including head space) used in this study.

2.2. Additional electrochemical data

*2.2.1. Redox couples of **3***

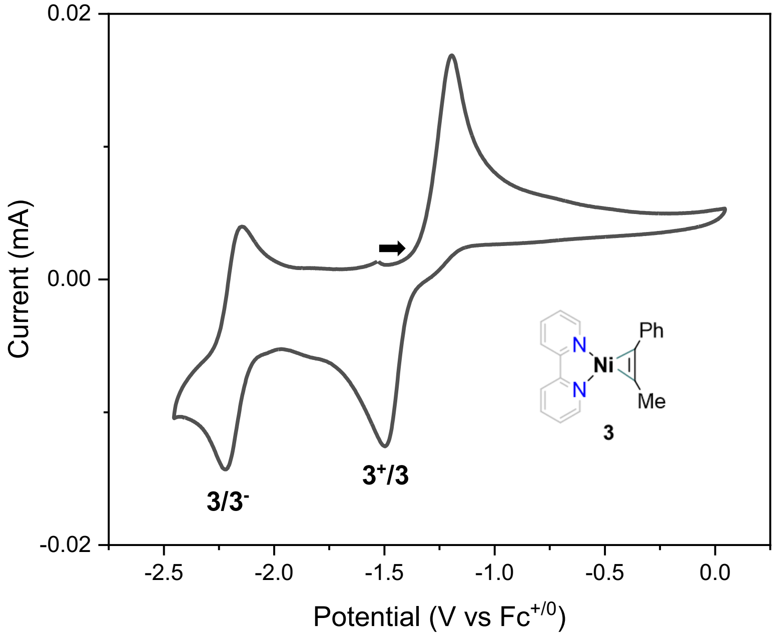

Figure S1. CV (oxidation first) of **3**.

*2.2.2. Comparison of redox behaviour of **1** and **4***

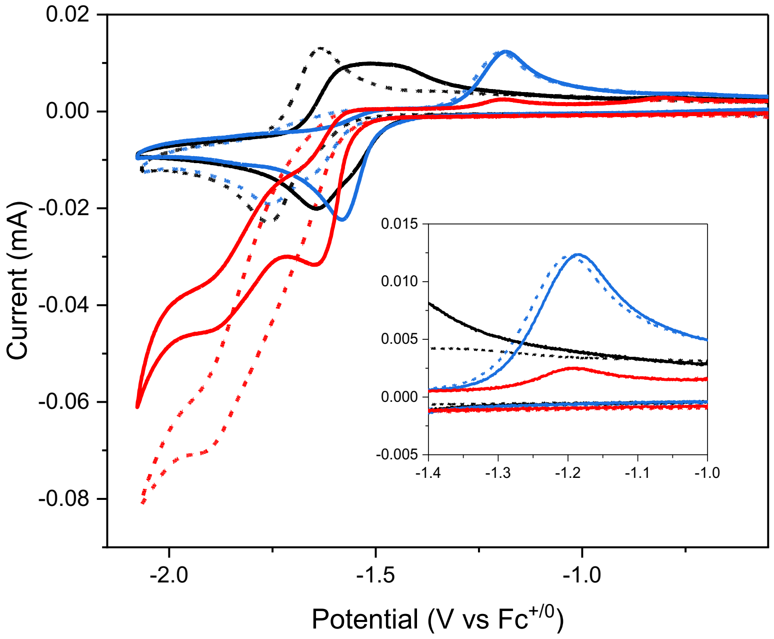

Figure S2. CVs of **1** (solid) or **4** (dashed) alone (black) or with **S1** (10 equiv) (blue) or with **S1** (10 equiv) and BzOH (50 equiv) (red).

Figure S2 shows the comparisons of CVs for the two precatalysts **1** and **4**. The reoxidation behavior ($E_{p,a}$ = –1.19 V_{Fc}) in the presence of **S1** and in the catalytic conditions (**S1** and BzOH) are fully consistent with the nickelacyclopropene resting state **3** as described in the main text. Interestingly, a negative shift of the foot of the reduction wave of ca. 50 mV is observed when switching from **1** alone (or with **S1**) to the catalytic conditions (**1**, **S1** and BzOH). We propose that this behavior arises from the initiation required for **1** to enter the catalytic cycle (from a tris-bipyridine to a mono-bipyridine ligand set).

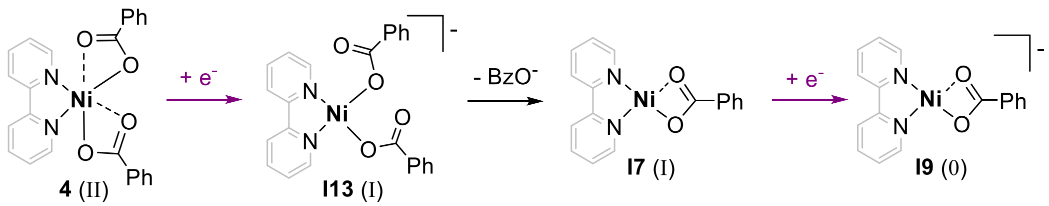

Scheme S1 . Reduction events associated with $4^{II/0}$.

*2.2.3. Behaviour of **4** in presence of benzoic acid*

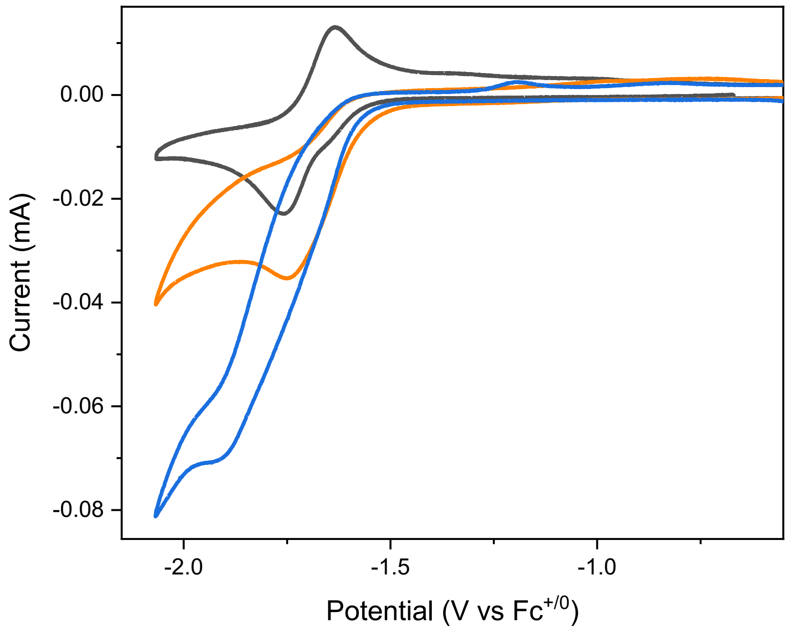

Figure S3. CVs of **4** alone (black) or with BzOH (50 equiv) (orange) or with **S1** (10 equiv) and BzOH (50 equiv) (blue).

Figure S3 shows the CV of **4** upon addition of equivalents of BzOH (with or without **S1** for comparison). The buildup of a minor electrocatalytic wave in the presence of BzOH only is ascribed to hydrogen evolution, as identified in an electrolysis experiment.

*2.2.4. Behaviour of **4** in presence of benzoate*

The voltammetry of **4** displays an ill-defined, broad reduction wave of $E_{p,c}$ = –1.76 V_{Fc}, with a shoulder at ca. –1.65 V_{Fc}. We thus here aim at clarifying the reduction behaviour of **4**.

First, CV of **4** with scan reversal right at the top of the shoulder (E_{rev} = –1. 67 V_{Fc}), which we designate as the first wave in the following, results in an irreversible wave (Figure S4a). This behavior is suggestive that this first wave could subscribe to an electrochemical reduction (E) followed by a fast chemical step (C), in an EC fashion. DFT calculations (see SI section 5.4.1) also indicate that the one-electron reduction product of **4** $[Ni(bpy)(BzO)_2]^-$ (**I13**) favors benzoate (BzO^-) expulsion to yield [Ni(bpy)(BzO)] (**I7**). In reason of the irreversibility of the first wave, we disfavor a CE mechanism, as such mechanism would in general display a reoxidation wave.

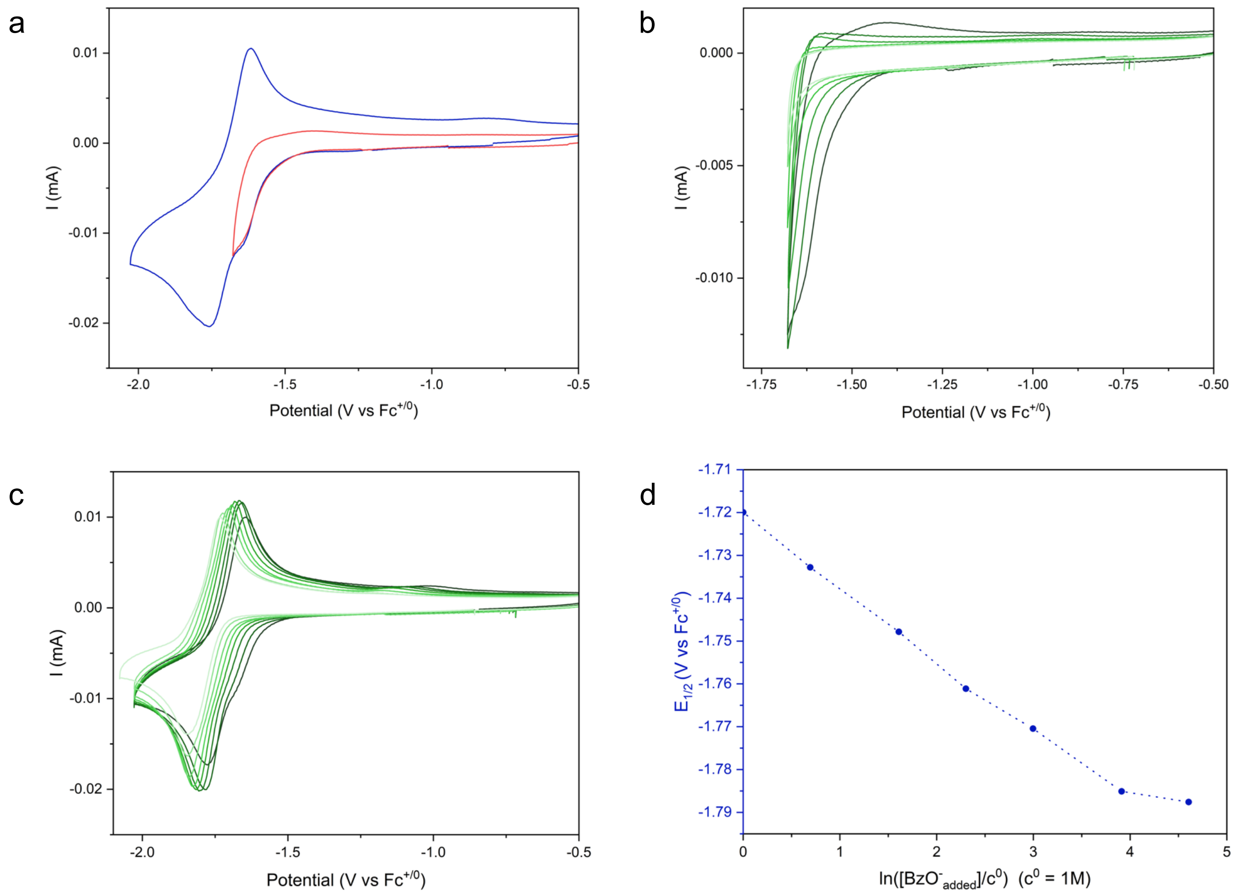

Figure S4. CVs of **4** a) alone and with increasing concentration of added BzO^- with scan reversal after b) the first wave and c) the second wave. From dark to light green: $[BzO^-]_{added}$ = 0, 1, 2, 5, 10, 20 mM. d) Apparent $E_{1/2}$ for the second wave as a function of $\ln([BzO^-]_{added}/C^0)$.

We thus tested the response of this wave towards BzO^- by incrementing concentration of this anion. We used for that DMF solutions of the $n$$Bu_4$NBzO salt, which features the same cation $n$$Bu_4N^+$ as our supporting electrolyte ($n$$Bu_4NPF_6$) and so that we can exclude additional cationic effects.

First, we observe on the CV of **4** that upon increase in $[BzO^-]$ (≤ 5 mM) the first wave shifts negatively (Figure S4b,c).

The influence of BzO^- on the first wave confirms that this electrochemical event is coupled to a chemical step involving BzO^-.

Increasing further [BzO⁻] (≥ 5 mM) the first reductive event vanishes under the second one in a wave having maximized peak intensity ($i_{p,c}$, $i_{p,a}$) magnitudes. The apparent $E_{1/2}$ (= ($E_{p,c}$+$E_{p,a}$)/2) of the resulting wave also displays a cathodic shift with increasing [BzO⁻] in a Nernstian fashion (Figure S4d).

From this body of data, we propose that, with no added BzO⁻ (Figure S5a), the second-order equilibrium between **I13** to **I7** is strongly displaced towards **I7**, with little influence of the backward association of BzO⁻. In this case, the first wave thus likely corresponds to the reduction of **4** to **I13**, followed by fast release of BzO⁻ to yield **I7**. Such a reduction scheme would subscribe to an EC mechanism, typically in the KP region of the kinetic zone diagram,[3] rather than a CE one. We also note that, at the potential of this first wave ($E_{p,c}$ ≈ –1.65 V_{Fc}, with no added BzO⁻) the resulting complex **I7** is likely not further reducible (into **I9**) and can only be reduced at more negative potentials, reached at the second wave. At the potential of the second wave ($E_{p,c}$ = –1.78 V_{Fc}, with no added BzO⁻) **I7** is reducible, leading to a second reduction event into **I9**. The reduction of **I9** is likely reversible on the CV timescale, which would corroborate the apparent reversibility of this wave. We thus propose that the ill-defined nature of the overall reduction pattern for **4** actually arises from two reductive events of close redox potentials, at low [BzO⁻]. An electrolysis of **4** (alone) negative to these waves (E_{app} = –2.1 V_{Fc}; data not shown) saturates current passed 2 mol(e⁻)/mol(**4**), which also confirms the 2-electron stoichiometry of the event.

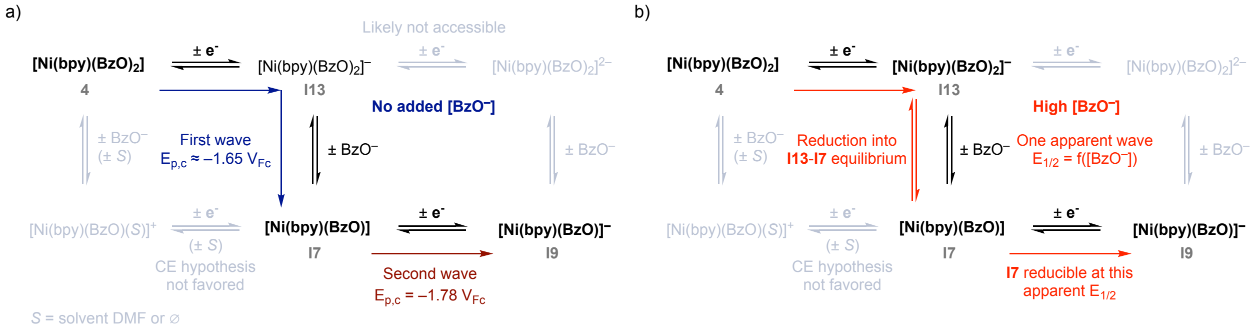

Figure S5. Proposed description of E and C steps involved in the reduction **4** with a) no added BzO⁻ and b) at high [BzO⁻].

At high BzO⁻ concentration (≥ 5 mM), we surmise that the influence of the backward association in the equilibrium between **I13** and **I7** cannot be neglected anymore (Figure S5b). There, **4** is plausibly reduced into an equilibrated mixture of **I13** and **I7** (assuming that both forward and backward rate constants are fast at voltammetric timescale). Under these conditions, the apparent redox potential of **4** would then be dictated by the equilibrium concentrations of **I13** and **I7**. At this apparent potential, further reduction into **I9** is likely accessible, giving rise to a better-defined two-electron reduction wave experiencing a Nernstian shift with [BzO⁻]. We cannot fully exclude that, at this apparent potential, reduction of **I13** (followed by BzO⁻ release) also becomes accessible.

We note that these interpretations of experimental data fall in good qualitative agreement with DFT results, which suggest that the reduction potentials of **4** and **I7** (E^0 = –1.59 and –1.76 V_{Fc}, respectively) are close but not inverted. In addition, DFT data (see Scheme S2) also corroborates that the equilibrium between **I13**, BzO⁻ and **I7** is moderately displaced in favor of the latter (by –2.8 kcal.mol⁻¹) and accessible ($\Delta\Delta G^‡$(**TS**$_{I13\text{-}I7}$) = 4.5 kcal.mol⁻¹), which, within

error of the computational methods, agrees with an equilibrated situation when [BzO^-] is built up, with fast forward and backward rate constants.

2.2.5. Additional mechanistic details

We discuss here the various plausible mechanistic pathways along the electrocatalytic wave and the influence of BzO^- on the electrocatalytic behaviour.

First, we stress that, under our experimental conditions, full conversion of the alkyne (10 mM) results in an expected BzO^- concentration of 20 mM. Thus, concentrations relevant for catalysis are $0 \leq [BzO^-] \leq 20$ mM. We more particularly focus our discussion to the cases with no to low concentration of added BzO^- as our kinetic analysis (viz. FOWA) is based on initial concentrations.

In the case where no BzO^- is added, we observe that the addition of the alkyne **S1** (10 mM) results in a positive potential shift of the first wave (Figure S6a). This shift is indicative of an EC mechanism involving **S1**, viz. the fast and irreversible formation of **I8**, as proposed in our main manuscript (Scheme 4). This point is further confirmed by scan reversal at the top of this wave, which evidences in the backward anodic scan the oxidation wave of the nickelacyclopropene **I8**. Upon gradual addition of BzOH, catalysis develops from this wave (Figure S6b). We thus attribute the activity on this wave to the mechanism initiating via **I7**, then **I8**, and following the ECEC-type pattern as described in our main manuscript.

When BzO^- is purposely added, the first electrocatalytic wave shifts towards more negative potentials (as observed for **4** alone), while the catalytic current at the potential of the second wave ($E \approx -1.78$ V_{Fc}) appears less affected (Figure S6c). At these more negative potentials, typically between –1.7 to –1.8 V_{Fc}, a pathway shuttling via the reduction of **I7** is also plausibly accessible. This pathway may not be the one dominating at the early stage of catalysis, where the one shuttling via **I8** is more favored. However, at late stage of catalysis when BzO^- concentration has built up in the cell, the pathway via **I8** likely shuts down and the one involving reduction of **I7** can become predominant. We thus tentatively assign the activity in this region of potential to the pathway for which **I7** is reduced to **I9**, then leading to substitution of BzO^- by **S1** into **3**, from which follows the catalytic cycle described in our manuscript.

We used the CV data recorded at [BzO^-] = 10 mM, which simulates a state of 50% conversion, to estimate kinetics when the catalytic cycle is shuttling via the reduction of **I9** to **3** (on the second wave). Applying FOWA (taking E^0 = E(**I7**/**I9**) = –1.76 V_{Fc} from the DFT value estimate), we obtained an estimate of the TOF_{max} at 4.75 10^5 s^{-1} and a span of ca. 9.8 kcal.mol^{-1}. These values are also within good agreement with the values expected from DFT for the ECEC-type mechanism described in our main manuscript.

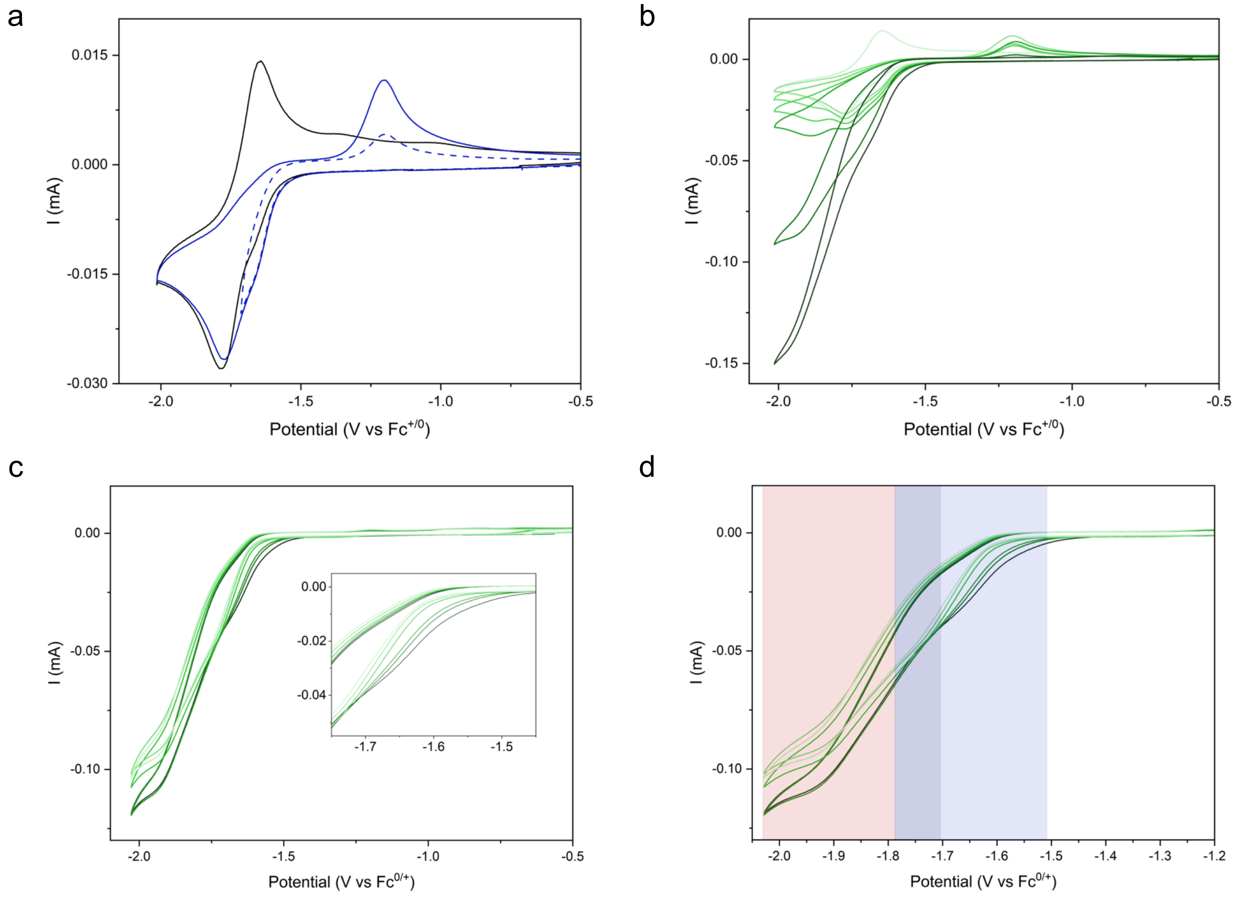

Figure S6. CVs of a) **4** alone (black) or with **S1** (blue), b) **4** alone (pale green) or with **S1** and increasing concentration of BzOH (from light to dark green: [BzOH] = 0, 1, 5, 10, 50, 100 mM) and c,d) **4**, **S1**, BzOH with increasing concentration of added BzO^{-} (from dark to light green: $[BzO^{-}]$added = 0, 1, 2, 5, 10, 20 mM) with in d) potential windows corresponding to the three catalytic regimes discussed below. Conditions: unless otherwise stated [**4**] = 1 mM, [**S1**] = 10 mM, [BzOH] = 100 mM.

Finally, the wave observed under electrocatalytic conditions at $E_{p,c} \approx -1.95$ V_{Fc} can likely be attributed to another mechanism, possibly implying the reduction of **I13** or the generation of a nickel hydride.

Overall, we propose that the electrocatalytic waves represent three main regimes, as schematically pictured on Figure S6d and summarized in Figure S7. At early stages of catalysis and potentials close to the foot-of-the-wave (Figure S6d, pale blue area), the pathway shuttling via **I8** (described in the main manuscript) is predominant. At advanced conversion leading to substantial $[BzO^{-}]$ in the cell and for intermediate potential values (ca. –1.7 to –1.8 V_{Fc}; Figure S6d, dark blue area), a pathway involving **I9** as intermediate to **3** is accessible and joins the catalytic cycle described in Scheme 4. At more negative potentials (< –1.8 V_{Fc}; Figure S6d, red area) another mechanism, for instance via the reduction of **I13** or a hydride, is also competing. The degradation of the FE toward alkene at more negative applied potentials along the electrocatalytic wave (Figure S8) supports that the ECEC-type mechanisms predominating close to the foot-of-the-wave have higher selectivity for alkyne semihydrogenation.

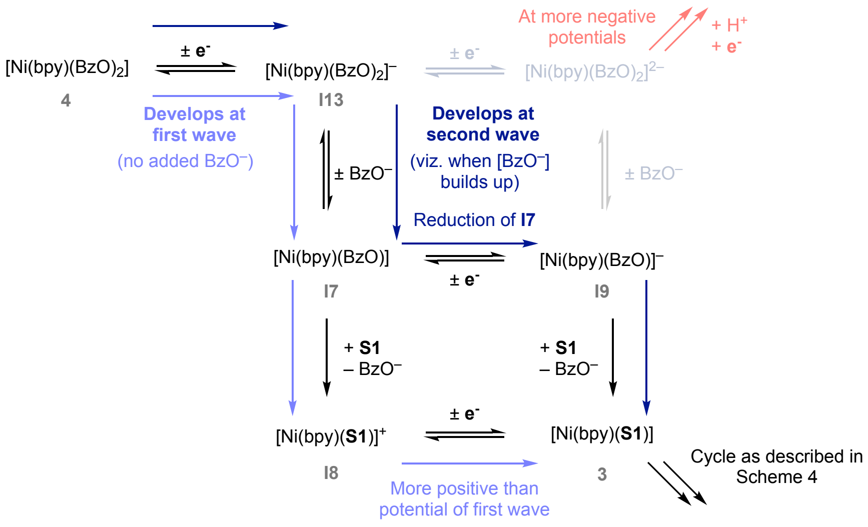

Figure S7. Proposed E and C steps involved for initiation to **3** at the first (via **I8**) and second (via **I9**) wave.

2.3. *Additional electrolysis results*

2.3.1. *Applied potential dependence*

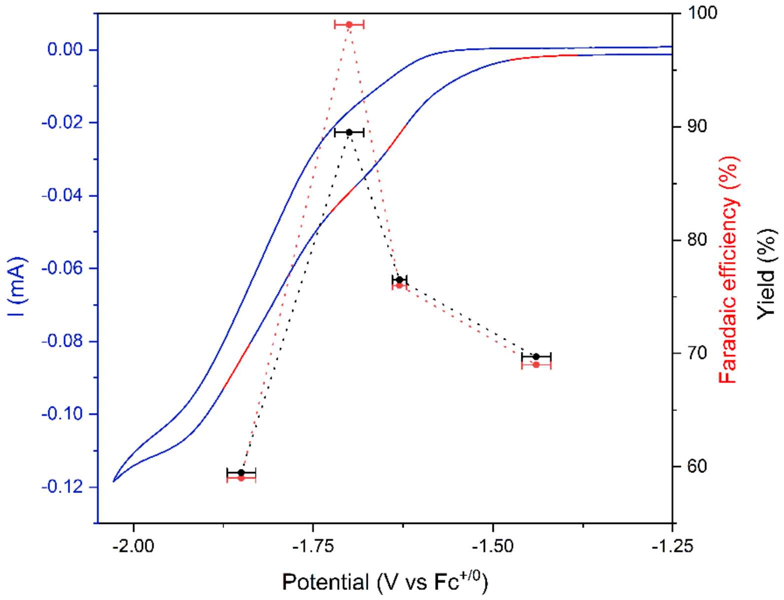

Figure S8. Faradaic efficiency (red) and olefin **S1H$_2$** yield (black) as a function of applied potential in electrolysis (dots, right scale) and CV of **4**, **S1**, BzOH (blue) with projections of the potentials applied in electrolysis (red areas) (left scale). Conditions: [**4**] = 1 mM; [**S1**] = 10 mM; [BzOH] = 100 mM; Q_{tot} = –9.7 C (= 2 mol(e$^-$)/mol(**S1**)); working electrode: C foam (electrolysis), glassy carbon electrode (CV). Electrolysis potentials corrected *a posteriori* for ohmic drop (i.R_{cell}; 100%); error bars represent the variations in i.R_{cell}-corrected potential due to fluctuation in current during electrolysis.

2.3.2. *Assessment of electrocatalytically active deposits*

The assessment of heterogeneous deposits responsible for the activity in electrocatalytic semihydrogenation was largely addressed in our previous manuscript[4] (especially in SI section 3.2.3.6). These results all suggest that, while we cannot fully rule out the deposition of (small) Ni species on the electrode, such species are minor and not

responsible for electrocatalytic activity. We also had run a control experiment using $[Ni(MeCN)_6]^{2+}$ as pre-catalyst and that showed little conversion (6% instead of full conversion with $[Ni(bpy)_3]^{2+}$ under identical conditions). This result further supported that the molecular nature of the catalyst and the presence of the supporting bpy ligands are integral to catalysis.

While these experiments were conducted with $[Ni(bpy)_3]^{2+}$ (**1**) as pre-catalyst, extension of this analysis to the $[Ni(bpy)(OBz)_2]$ (**4**) pre-catalyst is sensible, as the two pre-catalysts only differ in the initiation stage and further share the same catalytic pathway.

We reproduced here the "non-rinse" test with **4** since this test is a good reporter to evaluate the activity of any deposited species, whatever the nature of these species. We found that the current (Figure S9.) and activity (yield in **S1H$_2$**: 5.9 % vs 86.4 % for the first run including **4**) of the post-electrolysis, non-rinsed electrode are minor, which further supports that no catalytically active deposits form during electrolysis with **4**.

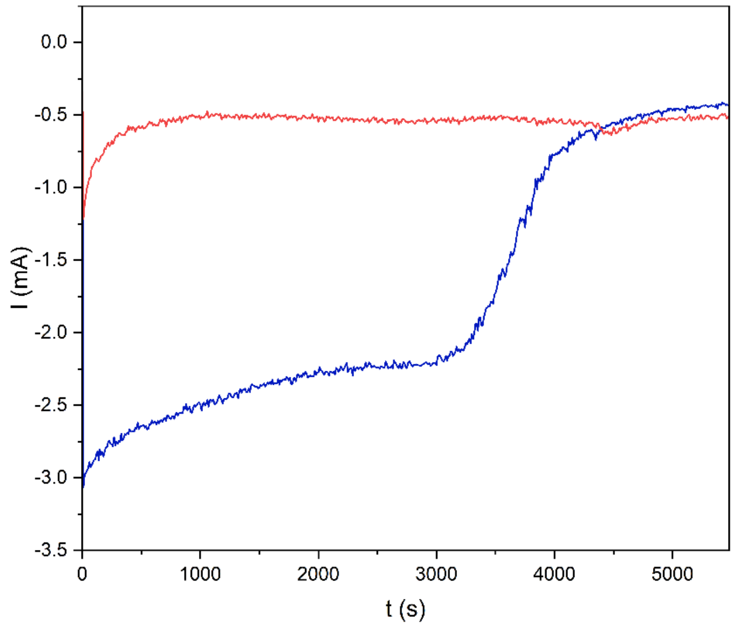

Figure S9. Current vs time during electrolysis with **4** (blue) and using the same electrode after removal of the electrolyte and replacement by an electrolyte exempt of **4** (red). Conditions: [**4**] = 1 mM (first run only); [**S1**] = 10 mM; [BzOH] = 100 mM.

In addition, we also performed an electrolysis using a Ni foam (purchased from *Goodfellow*; 1.6 mm thickness; 95% porosity) under conditions identical to with **4** (Figure S10). Under these conditions, alkyne consumption is below traces, whereas the use of **4** (at a glassy carbon foam) results in full conversion. These results underline that a Ni foam, which was reported active under other conditions, is not an active electrocatalyst under our conditions.

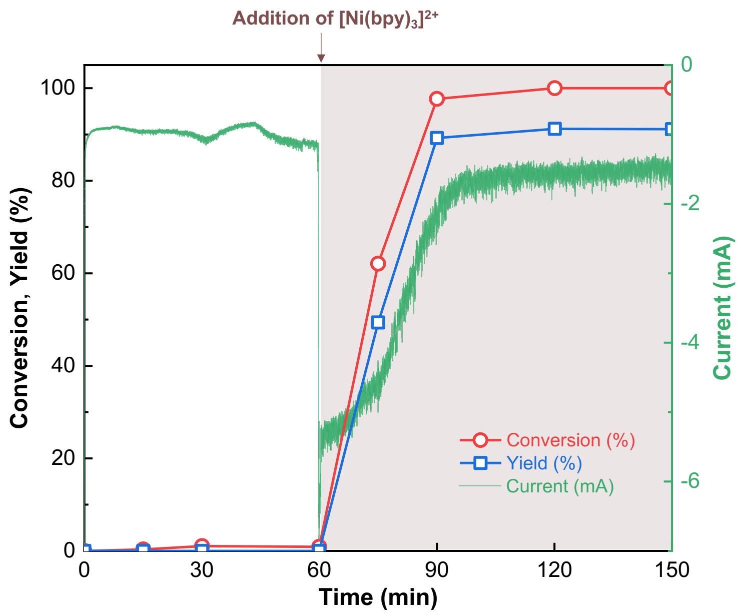

Figure S10. Current, conversion of **S1** and yield in **S1H$_2$** vs time during electrolysis at a Ni foam (white area) and upon addition of **1** (beige area). Conditions: [**1**] = 1 mM (second part only); [**S1**] = 10 mM; [BzOH] = 100 mM; DMF; 0.1 M nBu$_4$NPF$_6$; E_{app} = –1.7 V_{Fc}.

Interestingly, we also observed that the addition of the molecular pre-catalyst **1** in the electrochemical cell during electrolysis with Ni foam restores the activity. This result further proves the requirement of such molecularly-defined species for catalysis. The tolerance of the system for various electrode surface (C foam; Ni foam; Ag foil, data not shown) is also an additional indication that catalysis is driven by molecular species.

3. Spectroscopic characterizations

*3.1. Synthesis of [Ni(bpy)$_2$] (**2**)*

[Ni(COD)$_2$] (137.5 mg, 0.50 mmol, 1.00 equiv) and 2,2-bipyridine (156.2 mg, 1.00 mmol, 2.00 equiv) were introduced in a Schlenk tube in the glovebox and 5 mL of THF were added. The resulting deep blue solution was stirred 2 h at room temperature. The solvent and COD were evaporated under prolonged exposure to vacuum (10^{-3} mbar). The obtained solid was dissolved in THF (2 mL) and layered with pentane (10 mL). After 18 h, the solution was carefully removed from the solid and washed with pentane (3 x 10 mL) and dried under high vacuum (10^{-3} mbar) to afford 97 mg of **2** as dark shiny plates (yield = 52%).

The NMR data (^{1}H, ^{13}C; Figure S11, Figure S12) are in agreement with the literature.[5-6]

^{1}H NMR (400 MHz, THF-*d$_8$*): δ 10.17 (d, J = 5.9 Hz, 4H), 8.08 (t, J = 7.4 Hz, 4H), 7.70 (d, J = 8.4 Hz, 4H), 7.60 (t, J = 5.9 Hz, 4H). ^{13}C NMR (101 MHz, THF-*d$_8$*): δ 148.6, 140.0, 124.5, 120.6, 119.9.

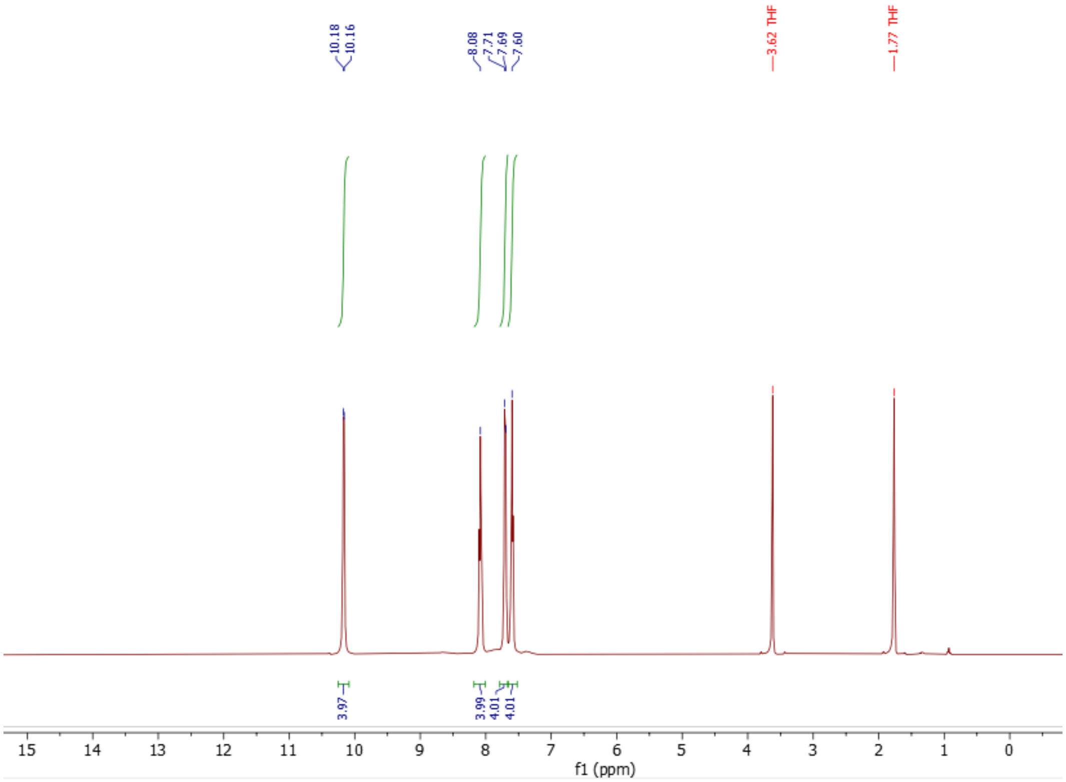

Figure S11. ^{1}H NMR (THF-*d$_8$*) spectrum of **2**.

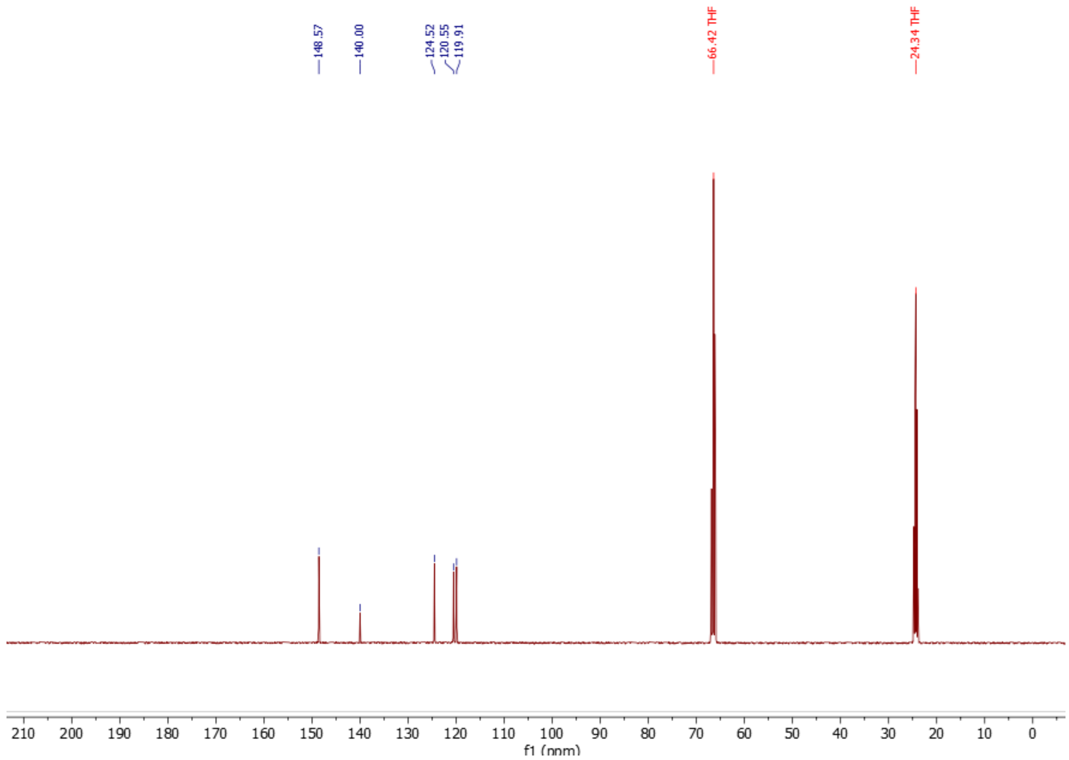

Figure S12. ^{13}C NMR (THF-d_8) spectrum of **2**.

3.2. Synthesis of [Ni(bpy)(PhCCMe)] (3)

The procedure is inspired from the reported synthesis of [Ni(bpy)(PhCCPh)].[2] [Ni(COD)$_2$] (137.5 mg, 0.50 mmol, 1.00 equiv) and 2,2-bipyridine (78.1 mg, 0.50 mmol, 1.00 equiv) were introduced in a Schlenk tube in the glovebox and 5 mL of THF were added. The resulting deep blue solution was stirred 2 h at room temperature and then kept in the freezer for at least 30 min at –30 °C. 1-ph-1-propyne (**S1**) is then added dropwise (62.6 µL, 0.55 mmol, 1.1 equiv) and the resulting red brownish solution was warmed up to room temperature and stirred overnight. This solution was then filtered over glass frit (P4 pore size) in glovebox and the solvent was evaporated under vacuum. The dark powder obtained was dissolved in THF (0.5 mL), layered with pentane (3 mL) and kept at –30 °C for a few hours. Small dark plates suitable for XRD were obtained. The solution was then carefully removed from the solid that was then washed with pentane (3 x 5 mL) and dried under vacuum (10^{-3} mbar) to afford 107 mg of **3** as a dark solid (yield = 65%). This compound is highly sensitive and degrades in a J. Young NMR tube after 12 h.

Elemental analysis found (calculated for $C_{19}H_{16}N_2Ni$): C: 68.7±1.2% (68.9%), H: 5.0±0.4% (4.9%), N: 8.2±0.4% (8.5%).

^{1}H NMR (400 MHz, THF-*d$_8$*): δ 10.17 (d, J = 5.5 Hz, bpy 6/6'), 10.10 (d, J = 5.5 Hz, bpy 6/6'), 8.00 (m, 4H, bpy 3/3' and 4/4'), 7.51 (t, J= 6,5 Hz, 2H, bpy 5/5'), 7.43 (d, J= 7.5 Hz, 2H, Ph ortho-H), 7.23 (t, J= 7.5 Hz, 2H, Ph meta-H), 7.04 (t, J= 7.5 Hz, 1H, Ph para-H), 2.60 (s, 3H, Me). ^{13}C NMR (101 MHz, THF-*d$_8$*): 152.4 (bpy 2/2'), 149.4, 148.9 (bpy 6/6'), 136.4 (Ph ipso), 131.5, 131.0 (bpy 4/4'), 127.7 (Ph ortho), 127.6 (Ph meta), 125.8 (bpy 5/5'), 123.5 (Ph para), 121.7, 121.6 (bpy 3/3'), 120.4 (C-Me,), 8.1 (Me); *C-Ph not observed*.

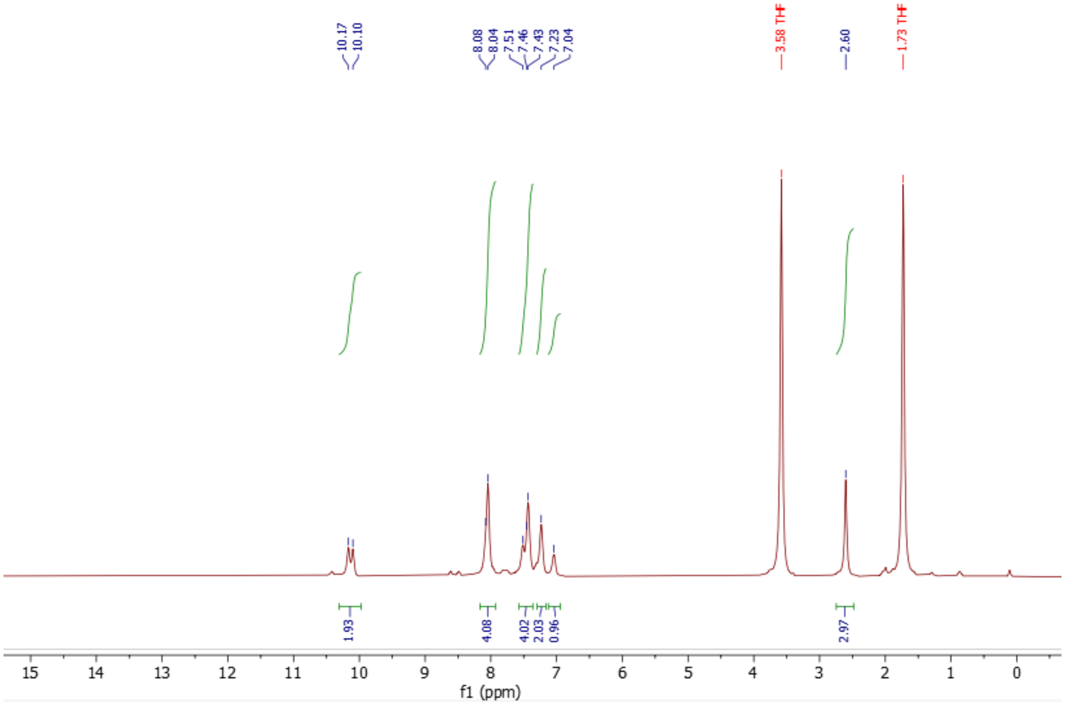

Figure S13. ^{1}H NMR (THF-*d$_8$*) spectrum of **3**.

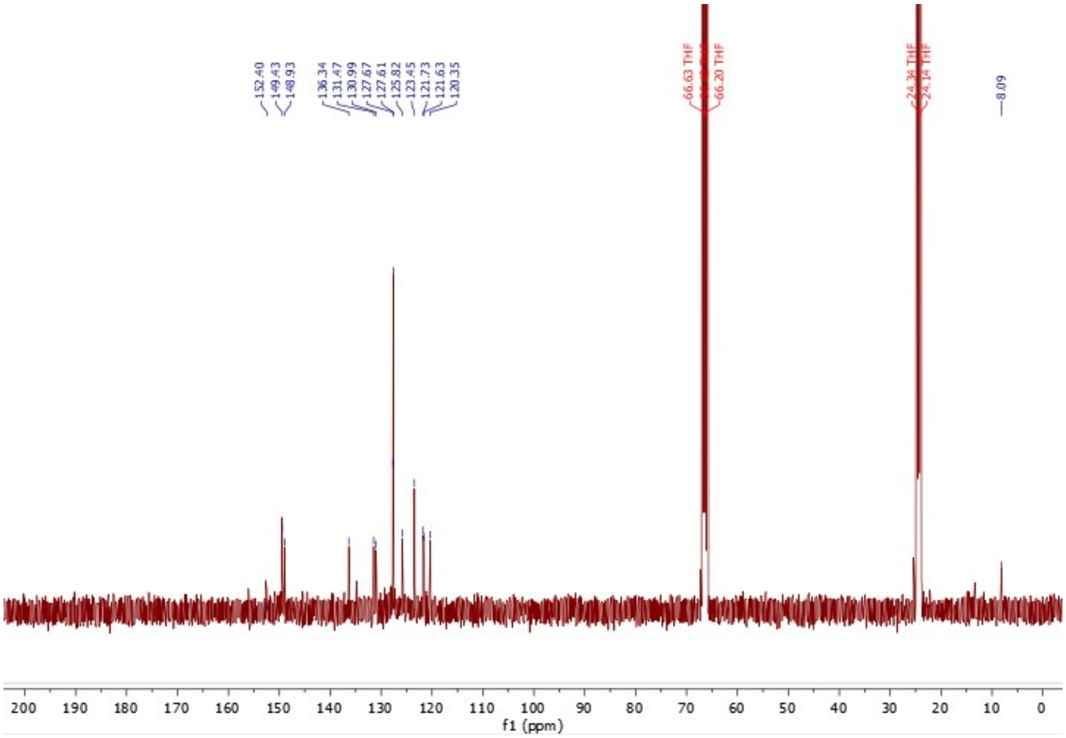

Figure S14. ^{13}C NMR (THF-d_8) spectrum of **3**.

3.3. *Synthesis of [Ni(bpy)(BzO)$_2$] (**4**)*

[Ni(bpy)(PhCCMe)] (**3**) (66.0 mg, 0.20 mmol, 1.00 equiv) and benzoic acid (48.8 mg, 0.40 mmol, 2.00 equiv) were introduced in a Schlenk tube in the glovebox and 5 mL of THF were added. The resulting grey blue-ish suspension was stirred 1 h at room temperature. The solvent was evaporated under vacuum and the obtained solid washed with THF (5 mL) and pentane (2 x 10 mL) and dried under high vacuum (10^{-3} mbar) to afford 58 mg of **4** as light blue powder (yield = 63%).

This solid is poorly soluble even in DMSO or DMF. However, we found that it is soluble in the presence of nBu$_4$NPF$_6$ in DMF (up to 4 mM, with a very slow solubilization which can be faster by using sonication or heat).

Elemental analysis found (calculated for $C_{24}H_{18}N_2NiO_4$): C: 62.6±1.2% (63.1%), H: 4.0±0.4% (4.0%), N: 6.0±0.4% (6.1%).

Alternatively, this compound can be synthesized from $NiCl_2$ according to the reported procedure.[7]

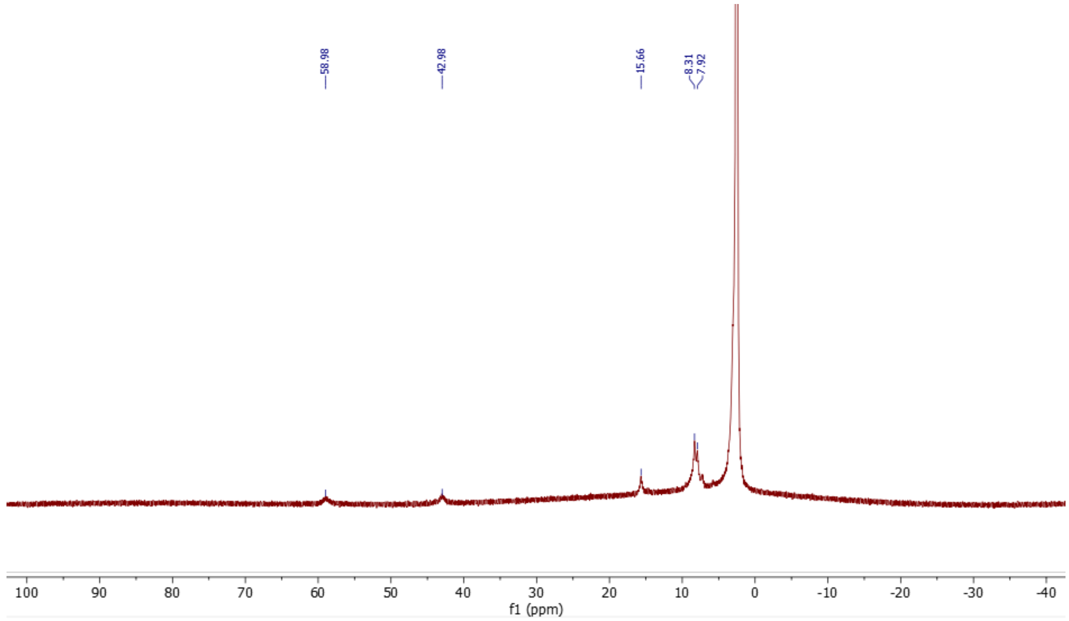

Figure S15. ^{1}H NMR (DMSO-d_6) spectrum of **4** (paramagnetic).

3.4. Stoichiometric experiments

*3.4.1. [Ni(bpy)$_2$] (**2**) + 1-ph-1-propyne (**S1**)*

2 (3.7 mg, 10 µmol) was dissolved in 0.5 mL of THF-*d*$_8$ in a J. Young NMR tube in the glovebox. **S1** (1.1 µL, 10 µmol, 1.0 equiv) and mesitylene as the standard (7.0 µL, 50 µmol) were added at r.t. The solution turned instantaneously dark brown-ish and the ^{1}H NMR spectra shows quantitative formation of **3** together with one free bpy ligand (Figure S16).

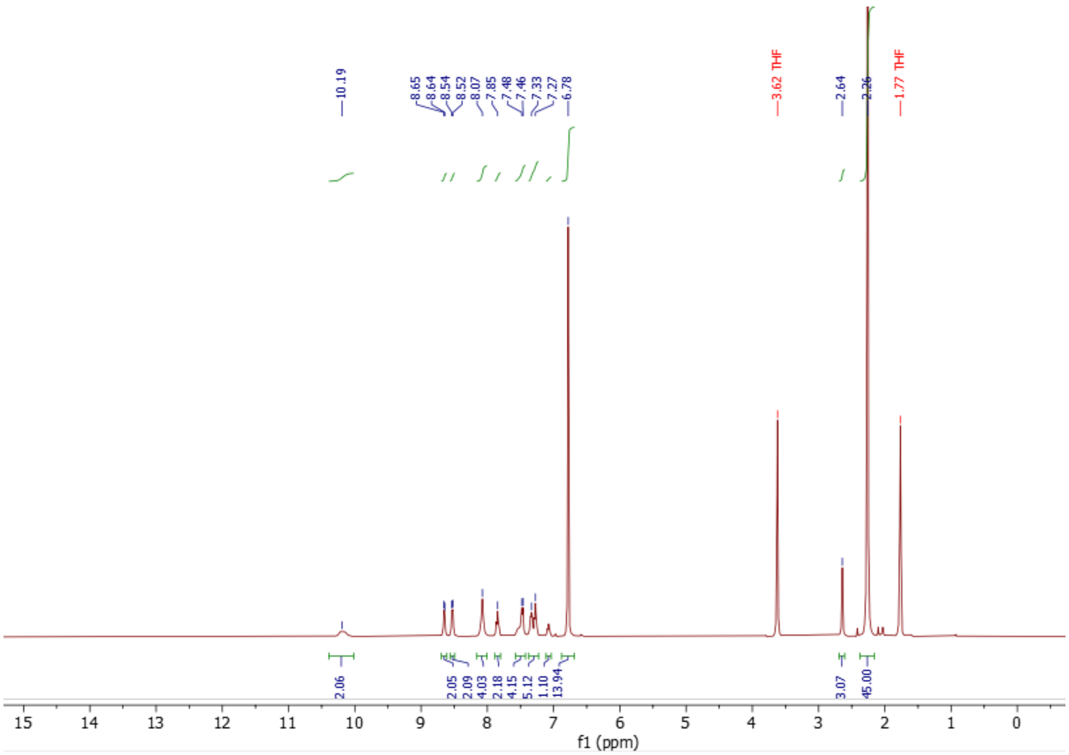

Figure S16. ^{1}H NMR (THF-*d*$_8$) spectrum of the crude mixture of **2** and **S1** after 5 min.

*3.4.2. [Ni(bpy)(PhCCMe)] (**3**) + benzoic acid (BzOH)*

3 (3.3 mg, 10 µmol) and *n*Bu_4NPF_6 (19 mg, 50 µmol) were dissolved in 0.5 mL of DMF-d_7 in a J. Young NMR tube in the glovebox and kept for at least 30 min at –30 °C. BzOH (1.2 mg, 10 µmol, 1.0 equiv) and mesitylene as the standard (7 µL, 50 µmol) were added and the mixture was allowed to warm up to r.t.

The ^{1}H NMR spectrum shows the appearance of two quadruplet signals at 5.0 and 5.3 ppm (J = 6.5 and 6.7 Hz respectively) in ca. 15% yield (Figure S17), which we attribute to vinylic protons, as these signals are close to the ones observed for a reported nickel(II) vinyl complex.[8] The absence of singlet signals suggest that the product of protonation in α-phenyl position is not formed and that the protonation is thus α-methyl regioselective. The two quadruplets are attributed to the two stereoisomers **I2** and **I2E** in a ratio of 8:2. The methyl protons of **I2** were also observed, at δ(^{1}H) = 2.8 ppm (J = 6.7 Hz). Despite repeated attempts, we were not able to isolate these species for further characterization. In the absence of *n*Bu_4NPF_6, these signals were not observed and only the alkene products were obtained (see below).

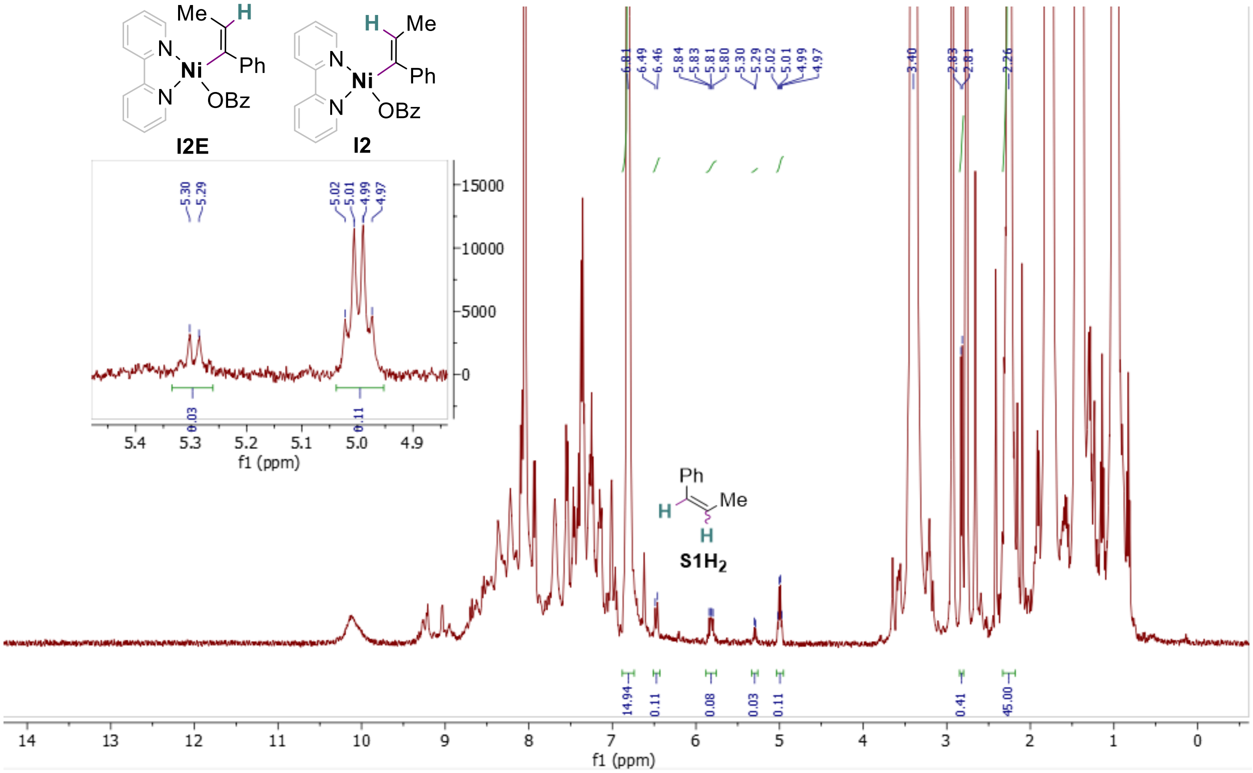

Figure S17. ^{1}H NMR (DMF-d_7) spectrum of the crude mixture of **3** and BzOH (1 equiv) after 5 min.

*3.4.3. [Ni(bpy)(PhCCMe)] (**3**) + 2 BzOH*

3 (3.3 mg, 10 µmol) and BzOH (2.4 mg, 20 µmol, 2.0 equiv) were dissolved in 0.5 mL of DMF-d_7 in a J. Young NMR in the glovebox at r.t. and mesitylene was added as the standard (7.0 µL, 50 µmol). A grey blue-ish precipitate is directly obtained that corresponds to **4** (checked by ^{1}H NMR in DMSO-d_6, see above SI section 3.3).

The ^{1}H NMR spectrum (recorded after few hours to let the precipitate sediment, Figure S18) shows the formation of the alkenes products (*Z*)- and (*E*)-stilbene in the *Z*/*E* ratio of 8:2 within a relatively high uncertainty, likely due to the presence of paramagnetic [Ni(bpy)(BzO)$_2$] (**4**) in solution that prevents good shimming.

This last effect also prevents the quantitative analysis by ^{1}H NMR of this reaction in the presence of nBu$_4$NPF$_6$ because of the solubilization of **4** in solution.

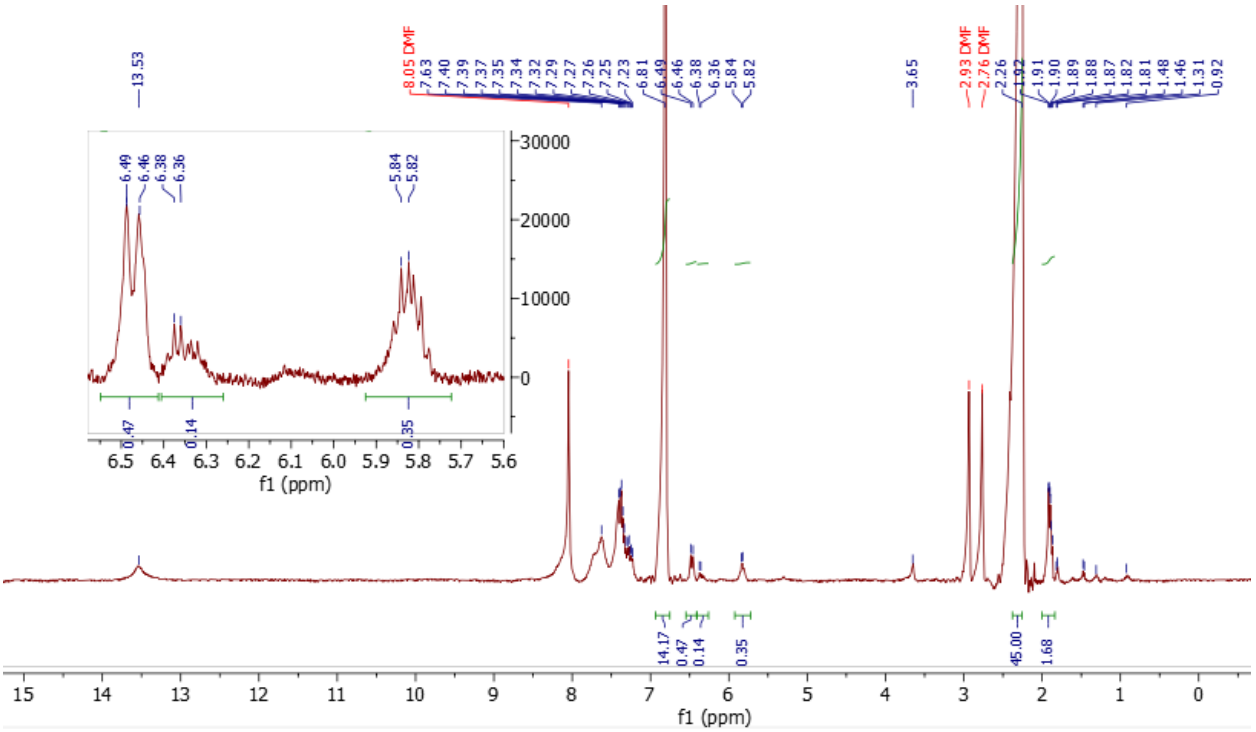

Figure S18. ^{1}H NMR (DMF-d_7) spectrum of the crude mixture of **3** and BzOH (2 equiv).

*3.4.4. [Ni(bpy)$_2$] (**2**) + BzOH*

2 (11.1 mg, 30 µmol, 3.0 equiv) and BzOH (1.2 mg, 10 µmol, 1.0 equiv) were dissolved in 0.5 mL of THF-d_8 in a J. Young NMR tube in the glovebox at r.t. and mesitylene was added as the standard (7.0 µL, 50 µmol). A grey blue-ish precipitate is directly obtained that corresponds to **4** (checked by ^{1}H NMR in DMSO-d_6, see above SI section 3.3). The ^{1}H NMR spectrum shows the formation of H_2 in ca. 70% yield (Figure S19, Figure S20).

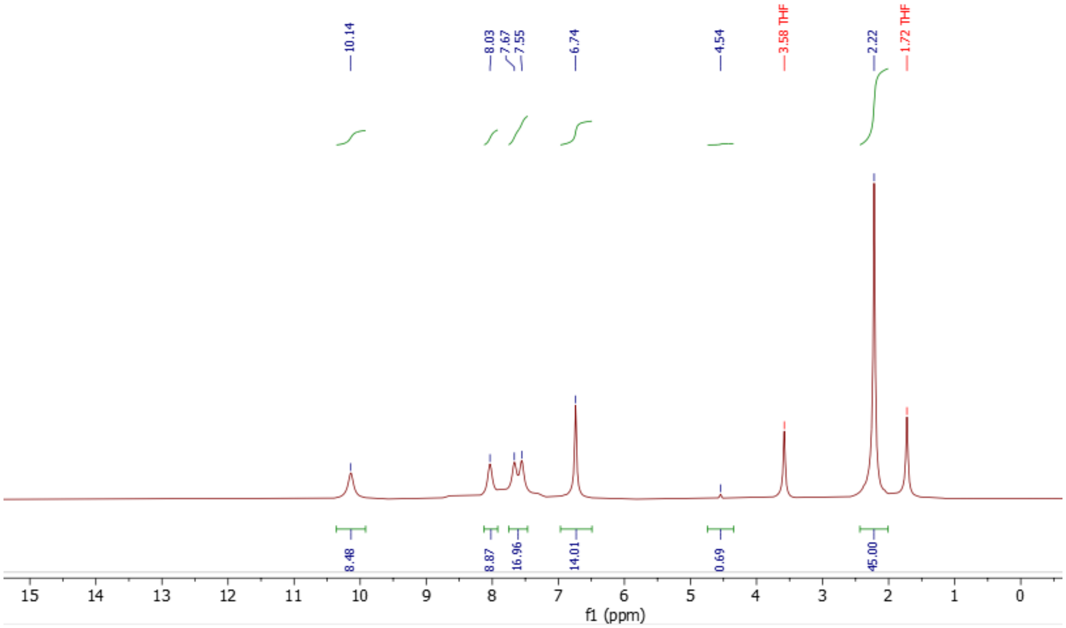

Figure S19. ^{1}H NMR (THF-d_8) spectrum of the crude mixture of **2** and BzOH (1 equiv) after 5 min.

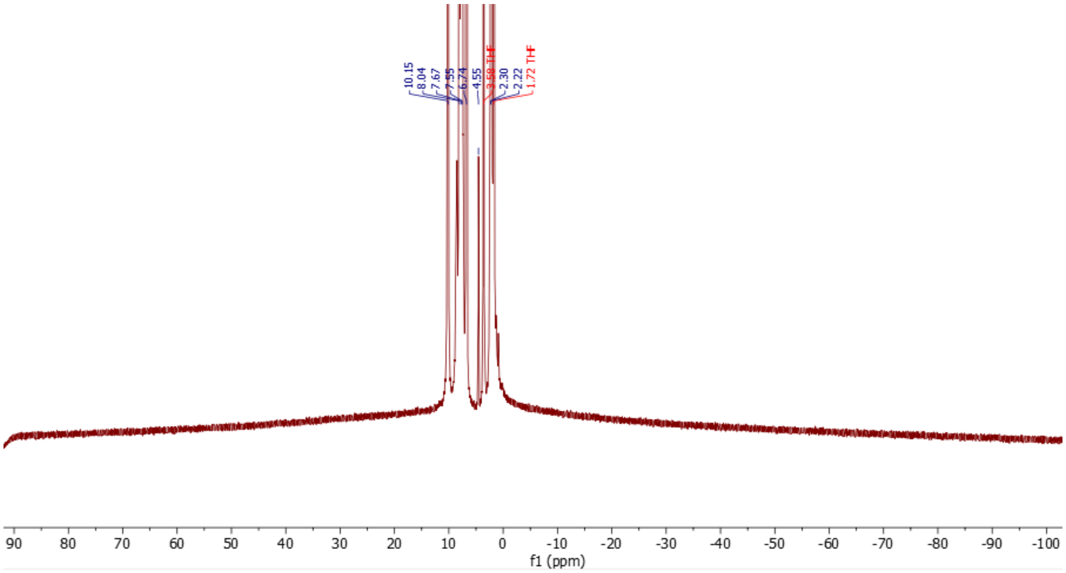

Figure S20. Wide range ^{1}H NMR (THF-d_8) spectrum of the crude mixture of **2** and BzOH (1 equiv).

We note that the wide range ^{1}H NMR spectrum of the mixture of **2** with one equivalent of BzOH does not display signals characteristic of a hydride species at Ni (Figure S20).

3.4.5. *[Ni(bpy)(PhCCPh)]* (**5**) *+ BzOH*

[Ni(bpy)(PhCCPh)] (**5**) (3.9 mg, 10 µmol) and *n*Bu_4NPF_6 (38 mg, 100 µmol) were dissolved in 0.5 mL of DMF-d_7 in a J. Young NMR tube in the glovebox and kept for at least 30 min at –30 °C. BzOH (1.2 mg, 10 µmol, 1.0 equiv) was added and the mixture was allowed to warm up to r.t. (Figure S21).

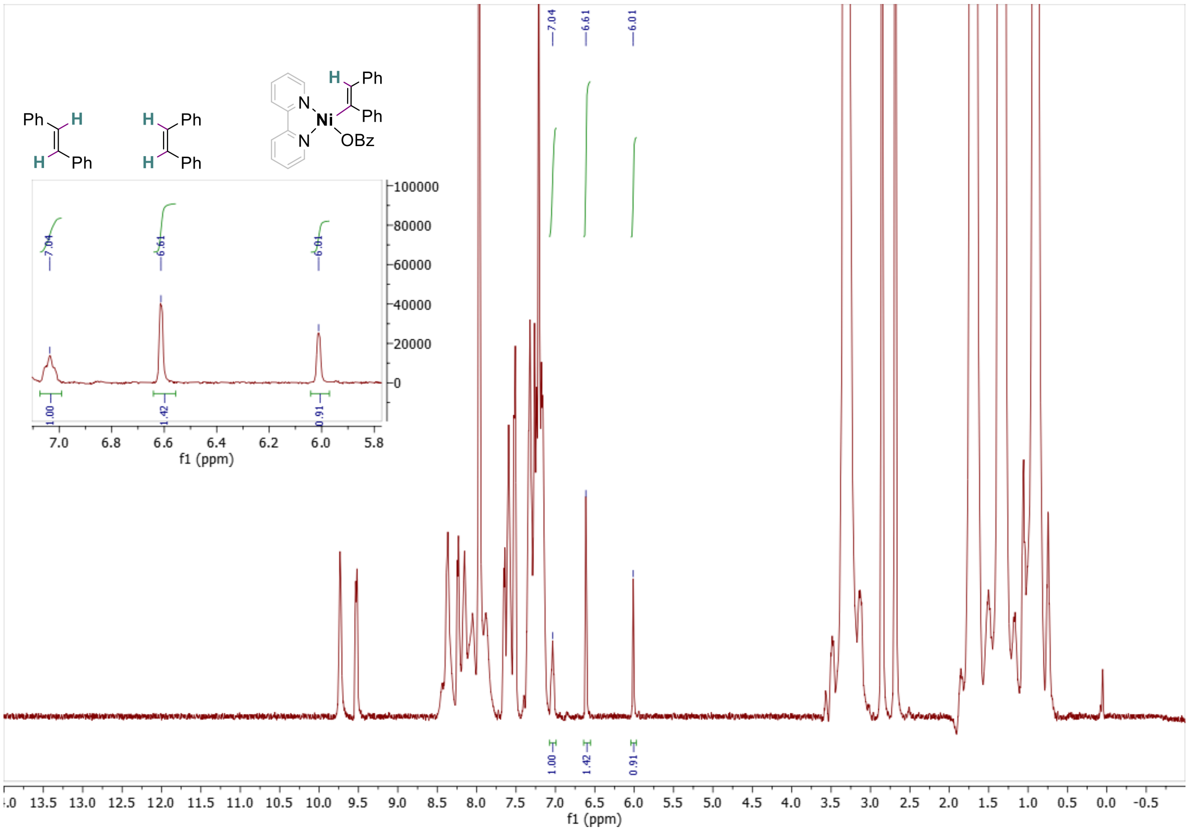

Figure S21. ^{1}H NMR (DMF-d_7) spectrum of the crude mixture of [Ni(bpy)(PhCCPh)] and BzOH (1 equiv) after 5 min.

The ^{1}H NMR spectrum shows the appearance of a singlet at 6.01 ppm (Figure S21), which we tentatively assign to the corresponding [Ni(bpy)(PhCCHPh)(BzO)] vinyl intermediate. In that case, only one isomer is detected, which is likely the Ni-*cis*-protonated one, by comparison with the shift of the free (*Z*)-olefin and in agreement with the shift reported for a similar stilbene Ni(II)-vinyl complex.[8] Interestingly, the ^{1}H NMR also reveals the evolution of the two isomers of the olefin product in a *Z/E* 59:41 ratio. Based on these results, we surmise that both vinyl isomers are accessible, but only the (*Z*) one is stable enough for trapping and observation on the experimental timescale, while the (*E*) one is less stable and readily converts to the corresponding olefin. Such a trend in relative stability of the vinyl isomers is also evidenced by computation on the Ni-vinyl isomers evolved using the 1-phenyl-1-propyne **S1** substrate (see Scheme 4 in main manuscript).

3.4.6. *Electrochemical stoichiometric experiments*

The formation of **3** under electrochemical conditions was conducted in the glovebox. The electrolysis was run with $[Ni(bpy)_3](BF_4)_2$ (**1**;2 BF_4^-) (10 mM) and 1-ph-1-propyne (**S1**; 100 mM) at E_{app} = –1.73 V_{Fc} until the current shows saturation (Q_f = –7.55 C; ca. 1.6 mole(e^-)/mole(**1**); 20 min). Then, 0.4 mL aliquots of the electrolytic solution were introduced in J. Young NMR tubes containing or not BzOH in benzene-d_6 (total volume of 0.5 mL in the NMR tube). In the absence of BzOH, the ^{1}H NMR spectrum namely shows two broad singlets at 10.14 and 10.08 ppm (Figure S22), in very good match with the ones observed for chemically synthesized **3** (see above SI section 3.2). In the presence of BzOH (1 or 2 equiv) the ^{1}H NMR spectra show signals of the **S1H$_2$** alkene in a *Z*/*E* ratio of 8:2 (Figure S23). When only one equivalent of BzOH is present, a quartet at 5.04 ppm (J = 6.5 Hz) can additionally be observed (Figure S24) and that we attribute to the Ni(II) vinyl complex **I2** (see SI section 3.4.2).

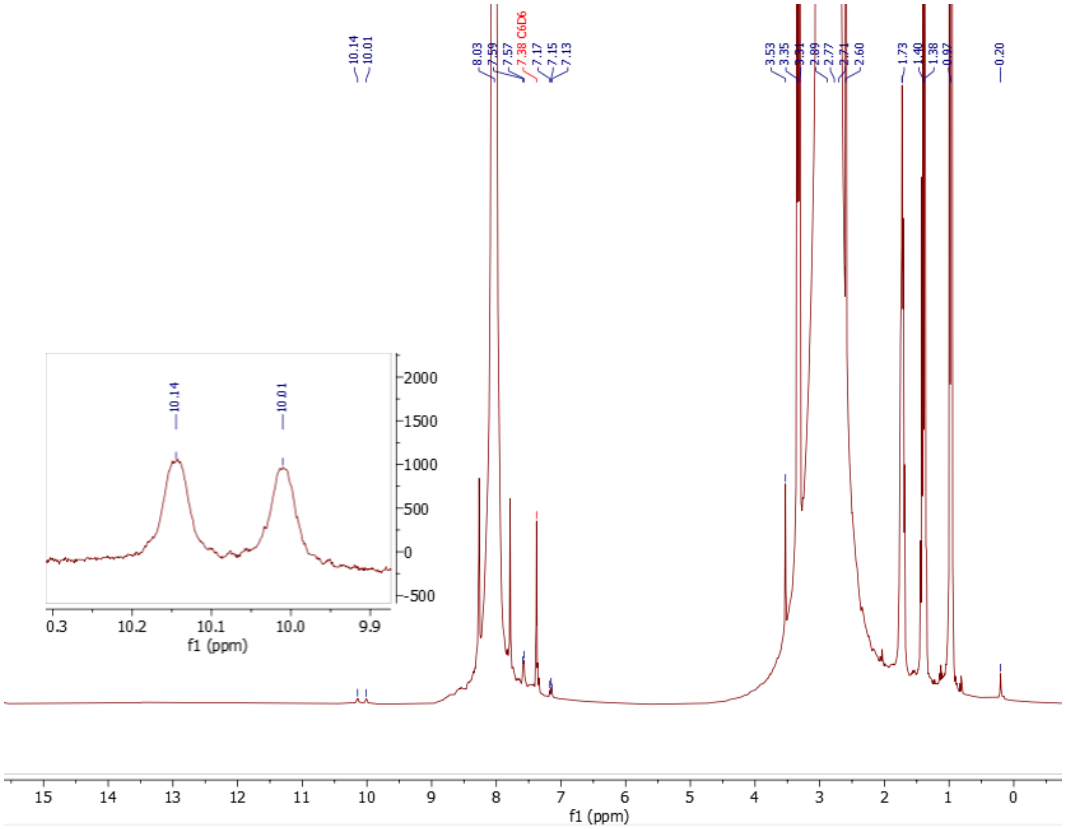

Figure S22. ^{1}H NMR (C_6D_6) spectrum of the post-electrolysis mixture, initially containing **1** and **S1**.

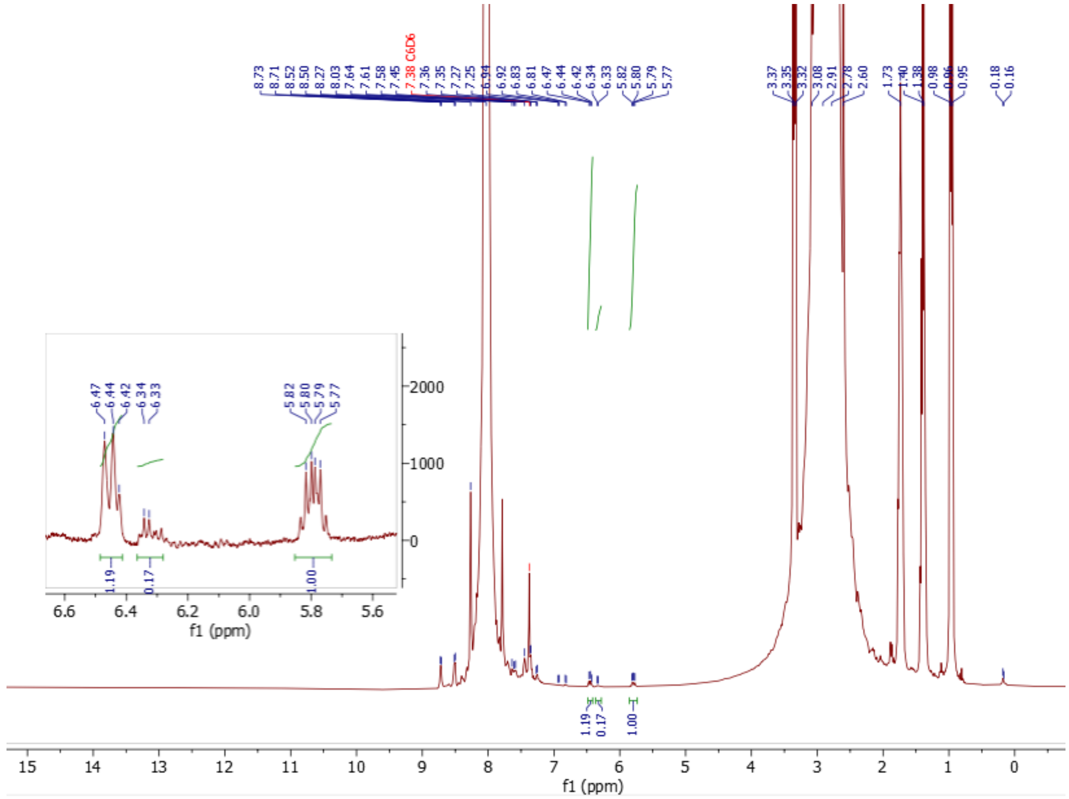

Figure S23. ^{1}H NMR (C_6D_6) spectrum of the post-electrolysis mixture, initially containing **1** and **S1**, after addition of BzOH (2 equiv).

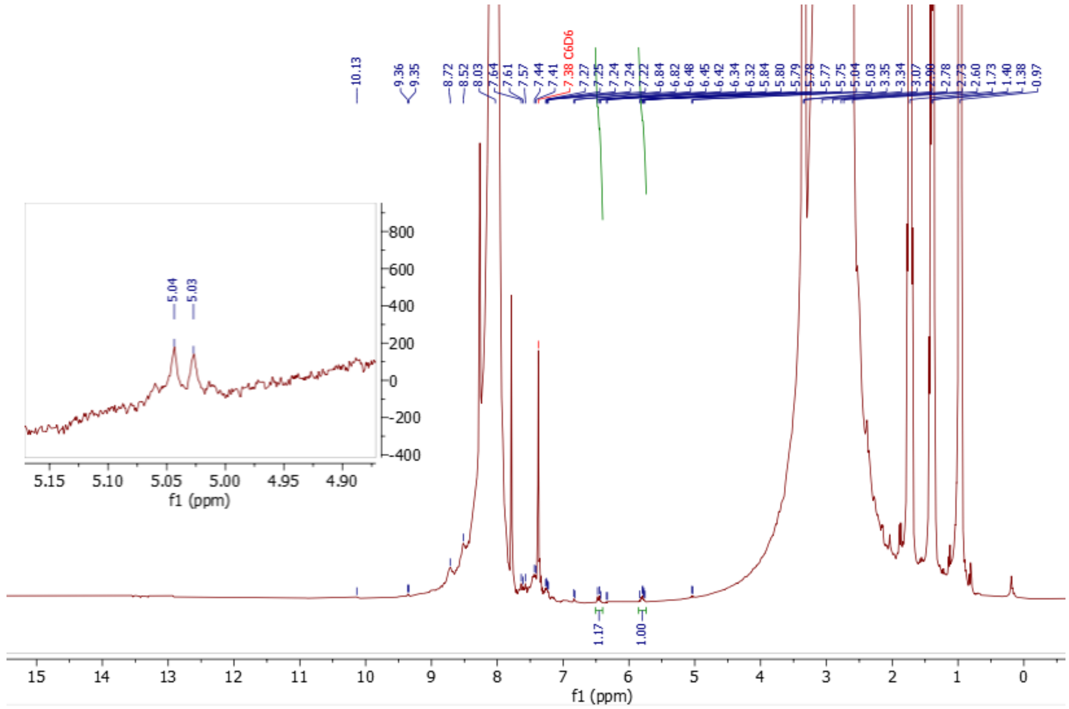

Figure S24. ^{1}H NMR (C_6D_6) spectrum of the post-electrolysis mixture, initially containing **1** and **S1**, after addition of BzOH (1 equiv).

3.5. XRD section

CCDC deposition	**2226437**
Molecular Formula	**C19 H16 N2 Ni**
Formula weight	**331.05**
Temp. (K)	**100(2) K**
Crystal system	**Orthorhombic**
Space group	**Pbca ; No. 61**
Absorption coefficient (mm^{-1})	**1.281**
a (Å)	**9.4957(5)**
b (Å)	**13.6021(7)**
c (Å)	**23.3963(12)**
α (deg)	**90**
β (deg)	**90**
γ (deg)	**90**
Z	**8**
V ($Å^3$)	**3021.9(3)**
Crystal dimensions (mm^3)	**0.100 x 0.100 x 0.015**
Abs. coeff. (mm^{-1})	**1.281**
Calc. density ($Mg{\cdot}m^{-3}$)	**1.455**
Wavelength (Å)	**0.71073**
F(000)	**1376**
h,k,l ranges collected	**-14<=h<=15, -14<=k<=22, -39<=l<=39**
θ range for data collection (deg)	**2.76 to 36.36**
Number of reflections collected	**103984**
Number of independent reflections	**7339 [R(int) = 0.0734]**
Completeness to theta = 25.24°	**99.9 %**
Absorption correction	**Semi-empirical from equivalents**
Max. and min. transmission	**0.98 and 0.81**
Data / restraints / parameters	**7339 / 0 / 200**
Refinement method	**Full-matrix least-squares on F^2**
Final R indices [I>2sigma(I)]	**R1 = 0.0400, wR2 = 0.0879**
R indices (all data)	**R1 = 0.0717, wR2 = 0.1028**
Goodness-of-fit on $F^{2\,c}$	**1.058**
Largest diff. peak and hole ($e.Å^{-3}$)	**0.574 and -0.458**

Table S1. Crystallographic parameters for **3**.

4. Foot-of-the-wave (FOWA) analysis

Kinetic data in this work are based on the foot-of-the-wave (FOWA) analysis developed by Savéant and coworkers[9] that enables extraction of the maximum turnover frequency (TOF_{MAX}) where the CV response is not affected by side phenomena. The proposed catalytic cycle is based on a mechanism of the type ECEC, as supported by DFT calculations (Scheme 4, main text) and for which equation (7) can be applied, but which is not restricted to this case (R is the perfect gas constant, T = 298 K and ν is the scan rate). i_p and $E°$ correspond to the peak current of a one-electron wave and the apparent half-wave potential of the catalyst **4** and were obtained from the CV of the catalyst in the absence of substrates.

$$\frac{i}{i_p} = \frac{2 \cdot 2.24\sqrt{\frac{RT}{F\nu}} \cdot \sqrt{TOF_{MAX}}}{1 + e^{\frac{(E - E°)F}{RT}}} \qquad \textbf{(7)}$$

We note that the assessment of the first reduction potential of **4** is obtained from an ill-defined experimental wave, which thus leads to uncertainties in the estimation of this value (E^0 = –1.64 V_{Fc}, at the experimental first wave), and that at high concentration of benzoic acid, homoconjugation phenomena[10] can impact the electrocatalytic answer. For FOWA at high [BzO^-], E^0 = –1.76 V_{Fc} was assumed from the DFT estimate of E(**I7**/**I9**).

We also point that the reduction of **4** is associated with an initiation process that only intervenes in the first catalytic cycle (Scheme 3Ab or Scheme S2). In general, the interplay of the initiation in the electrocatalytic wave complicates the electrochemical analysis. However, the initiation process pertains to initial catalytic conditions, at which the electrocatalytic wave develops and hence for which FOWA was applied.

The analysis of the values of TOF_{MAX} with varying concentrations of the different reaction partners allows to extract partial orders in each partner. The kinetic isotope effect, KIE, was determined by evaluating TOF_{MAX} for the reaction in the presence of the deuterated acid BzOD.

For higher accuracy, the currents of the CVs were normalized by subtracting the current from the blank CV performed at the beginning of each experiment. Kinetic analyses for alkyne, BzOH and BzOD (KIE) were also replicated with at least two different scan rates (0.05, 0.1 or 1 $V.s^{-1}$) to validate applicability and to provide uncertainty values.

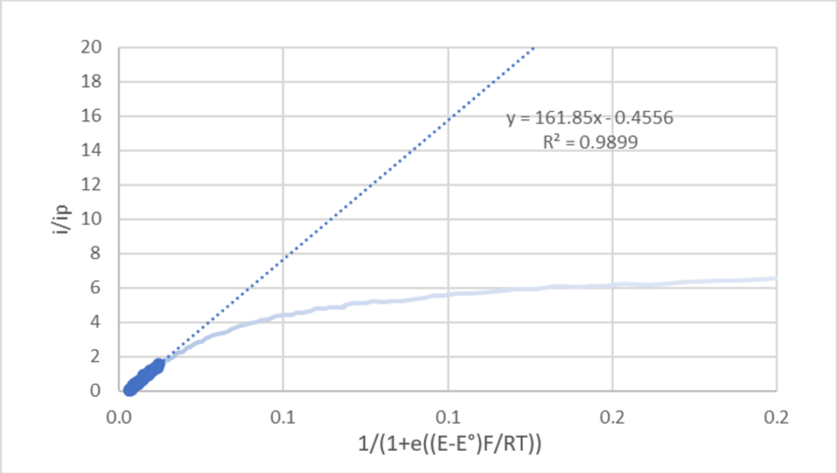

Figure S25. Representative example of data treatment for FOWA analysis.

5. Computational details

5.1. General consideration

Calculations were performed using the ORCA 5.0 suite of software.[11] The PBE0 functional[12] with Grimme's D3BJ dispersion correction[13] was used in conjunction with the 6-311+G(d,p) basis set for all atoms.[14-18] All geometries were fully optimized without any symmetry or geometry constraints. Harmonic vibrational analyses were performed to confirm and characterize the structures as minima or transition states. Free energies were calculated within the harmonic approximation for vibrational frequencies. The effects of the solvation by DMF were included in the energy calculations using the CPCM model.[19] Standard potentials were calculated with respect to the phenazine$^{0/–}$ redox couple and converted back versus the $Fc^{+/0}$ redox couple as previously described for accuracy.[20] The electron transfer steps were then calculated at –1.70 V relative to $Fc^{+/0}$ and are assumed to be faster than chemical steps.

5.2. Spin configurations

Unless otherwise stated, the spin configurations of the nickel complexes in this study are calculated in the singlet ground state and the doublet ground state for the open-shell nickel(I) complexes. The exceptions are for the known paramagnetic [Ni(bpy)(OBz)$_2$] (**4**) which was calculated as a triplet state and for the reduced nickelacyclopropene **3**$^{–I}$ where the experimental redox potential found for the **3**$^{0/–I}$ couple is much closer to the calculated quadruplet than to the doublet state: –2.13 (quadruplet) ~ –2.18 (experimental) ≠ –1.78 (doublet) V_{Fc} (see above SI section 2.2.1). Nevertheless, the UKS function implemented in ORCA was used for spin-unrestricted optimisation.

5.3. Computed KIE

Gibbs free energies (at PBE0-D3/6-311+G(d,p)//CPCM (Solvent: DMF) level of theory) of deuterated compounds BzOD and the deuterated TDTS **TS$_{3\text{-}I1D}$** (which derived from **TS$_{3\text{-}I1}$**) were obtained from single point calculations using the isotope option implemented in ORCA. **TS$_{3\text{-}I1D}$** is 11.3 kcal.mol^{-1} ($\Delta G_D^‡$) higher in energy than **3**. The rate-determining state **TS$_{3\text{-}I1}$** was 10.2 kcal.mol^{-1} ($\Delta G_H^‡$) higher in energy than **3**. The KIE was determined using the following equation (8):

$$KIE = k_H \ / \ k_D = e^{-(\frac{\Delta G_H^‡ - \Delta G_D^‡}{RT})} \qquad \textbf{(8)}$$

5.4. Additional computational results

5.4.1. Computed EECC pathway

Following the initial protonation step (C) of the mechanism presented in the main text, **I2** (–5.5 kcal.mol^{-1}) can be obtained. A second protonation step (C) on **I2** most likely proceeds in an outer-sphere manner (see SI section 5.4.3 for inner-sphere) with a relatively high energy barrier involving **TS$_{I2\text{-}I12}$** (+15.6 kcal.mol^{-1}) and gives the cationic species **I12** (–3.5 kcal.mol^{-1}) (Scheme S2). From **I12**, the (*Z*)-alkene product is readily released via **TS$_{I12\text{-}I11}$** (+4.4 kcal.mol^{-1}) and the obtained cationic benzoate **I11** (–10.1 kcal.mol^{-1}) can undergo barrierless coordination of a benzoate ligand to give the bis-benzoate complex **4** at –18.5 kcal.mol^{-1}. The latter can be reduced (E) under the

electrolysis condition according to the calculated standard potential of –1.59 V_{Fc} (>E_{app} = –1.70 V_{Fc}) to generate a square-planar anionic Ni(I) bis-benzoate complex **I13** at –23.9 kcal.mol^{-1}. Then, the release of a benzoate ligand occurs via **TS$_{I13-I7}$** (–19.4 kcal.mol^{-1}) giving the Ni(I) benzoate complex **I7** (–26.7 kcal.mol^{-1}). From **I7**, the closure of this catalytic cycle is as for the ECEC-type mechanism described previously.

An alternative pathway where **I7** is reduced to the Ni(0) benzoate **I9** (–15.2 kcal.mol^{-1}) can also be considered as the reduction potential computed for **I7** (–1.76 V_{Fc}) is close to the one applied in electrolysis (–1.7 V_{Fc}). The second reduction (E) can also occur earlier in this mechanistic scheme, for example from **I12** or **I11** at –1.41 and –1.48 V_{Fc}, respectively. In all cases, these alternative pathways converged on the Ni(I) benzoate complex **I7**.

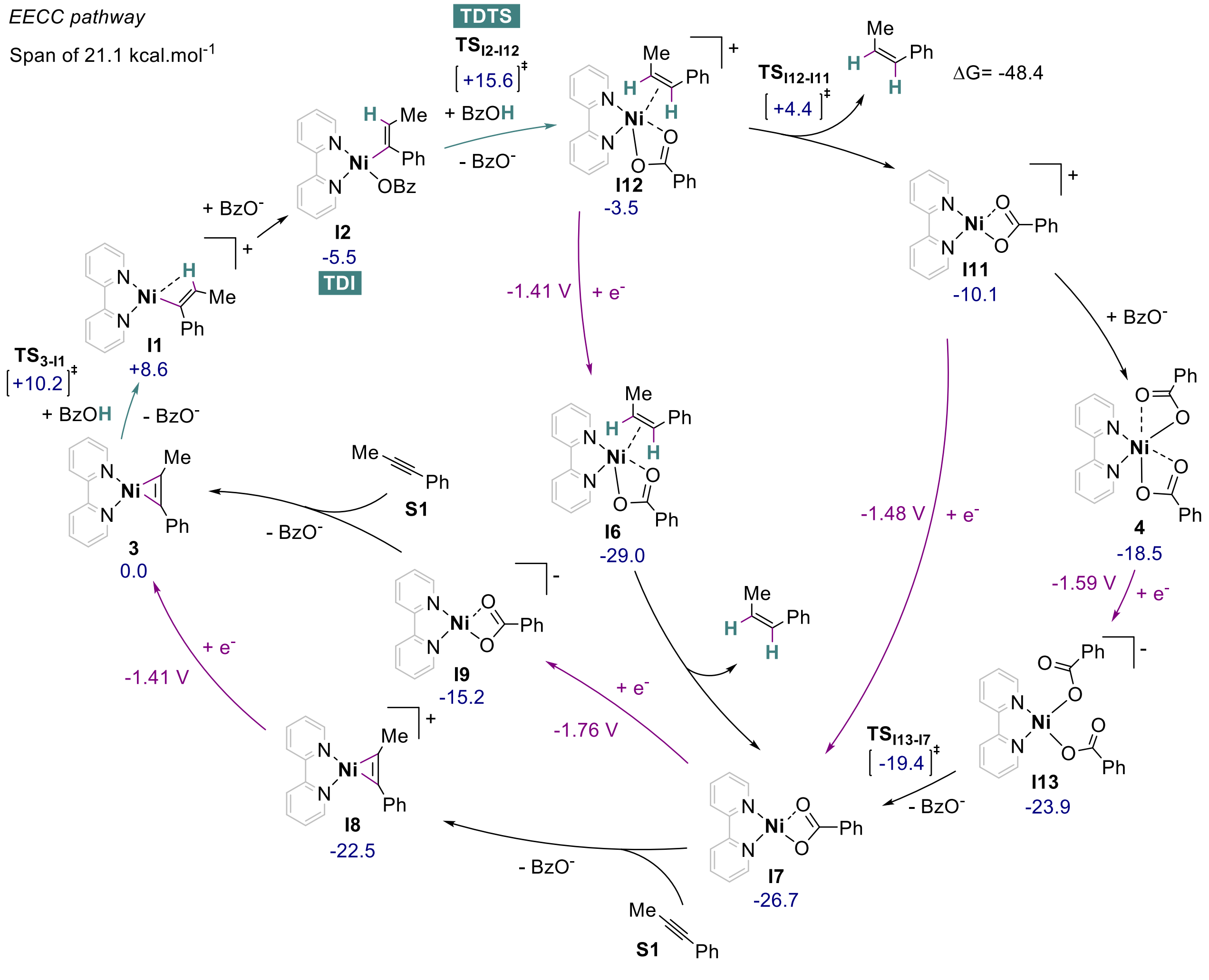

Scheme S2. Computed Gibbs energies for an EECC mechanism of the electrocatalytic semihydrogenation of alkyne **S1** with **3**. Conditions: PBE-D3/6-311+G(d,p) level of theory; CPCM model to account for solvent effect (DMF).

However, in all these cases, the energetic span of the reaction is defined by **I2** and **TS$_{I2-I12}$**, which corresponds to the second outer-sphere protonation step, and is found at a value of 21.1 kcal.mol^{-1}. This value is much higher than the span of 10.2 kcal.mol^{-1} associated to the ECEC-type mechanism (Scheme 4 in the main text). For that reason, we propose that, under electrocatalytic conditions, the ECEC-type mechanism is largely dominant over the EECC one.

Several other pathways were ruled out due to high energy values for intermediates or TSs, or reduction potentials of intermediates more negative than the applied potential of ca. –1.7 V_{Fc} (see SI section 5.4.3).

5.4.2. Hydride pathways

Scheme S3 represents other nickel hydrides that have been considered in the DFT calculations. In particular, the reduced form of **I10**, **I14** which is more stable. However, the transition states **TS**$_{\mathbf{I14\text{-}I3}}$ and **TS**$_{\mathbf{I14\text{-}I7}}$ corresponding to either hydrometallation or hydrogen evolution from **I14** display relatively high barriers for these two elementary steps ($\Delta\Delta G^{\ddagger}$ = +25.2 and +17.3 kcal.mol^{-1} respectively).

Despite repeated attempts to generate hydrido-vinyl Ni species, the only stable one that converged is the cationic Ni(IV) complex **I15**, which is though +32.9 kcal.mol^{-1} higher in energy than **3**. The corresponding Ni(III) hydride complex, which can be obtained by protonation of **I3**, was not found because the structure converges to **I6** during optimization.

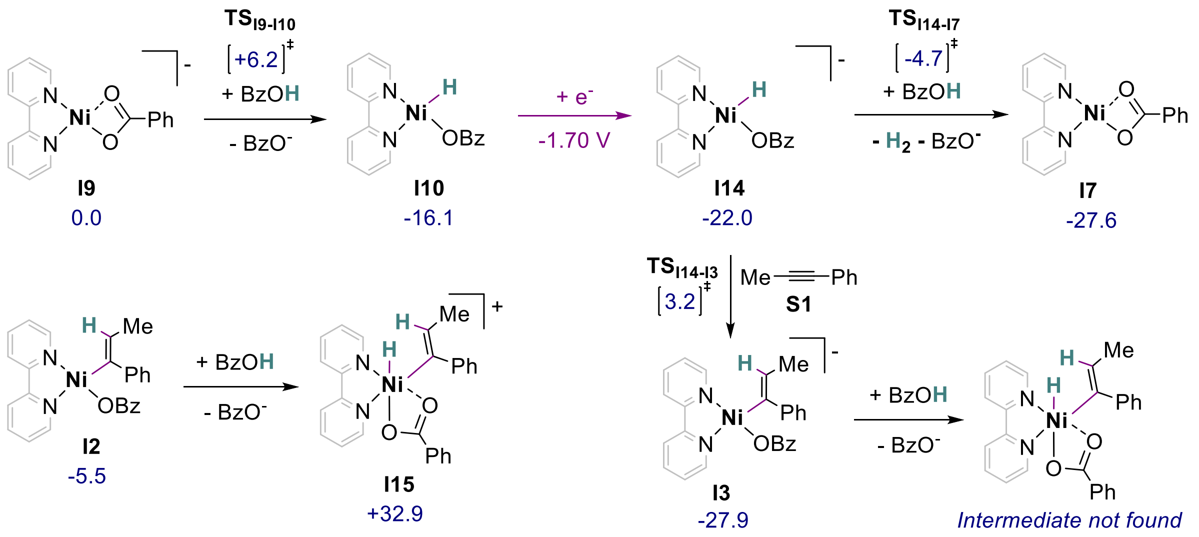

Scheme S3. Computed Gibbs energies for nickel hydride species. Conditions: PBE-D3/6-311+G(d,p) level of theory; CPCM model to account for solvent effect (DMF).

5.4.3. Stereoisomers, regioisomers and alternative transition states

Different pathways were studied to understand the stereoselectivity of the reaction (Scheme S4). From **3**, only the **TS**$_{\mathbf{3\text{-}I1}}$ transition state leading to **I1** was found. A possible TS leading to **I1E** in one step could not be found because during optimization, the geometry of **TS**$_{\mathbf{3\text{-}I1}}$ was always restored. After electron transfer, two species also in equilibrium **I4** and **I4E** are obtained.

The (*E*) isomers of the vinyl species **I2** and **I3**, i.e., **I2E** and **I3E**, were found to be thermodynamically more stable than the (*Z*) isomers, which does not reflect the observed stereoselectivity among olefin products. Furthermore, (*Z*)-to-(*E*) isomerization at **I2** is very unlikely, due to a very high transition state energy of **TS**$_{\mathbf{I2\text{-}I2E}}$ ($\Delta G^{\ddagger}$= +32.5 kcal.mol^{-1}). These latter selectivity-determining pathways were therefore excluded.

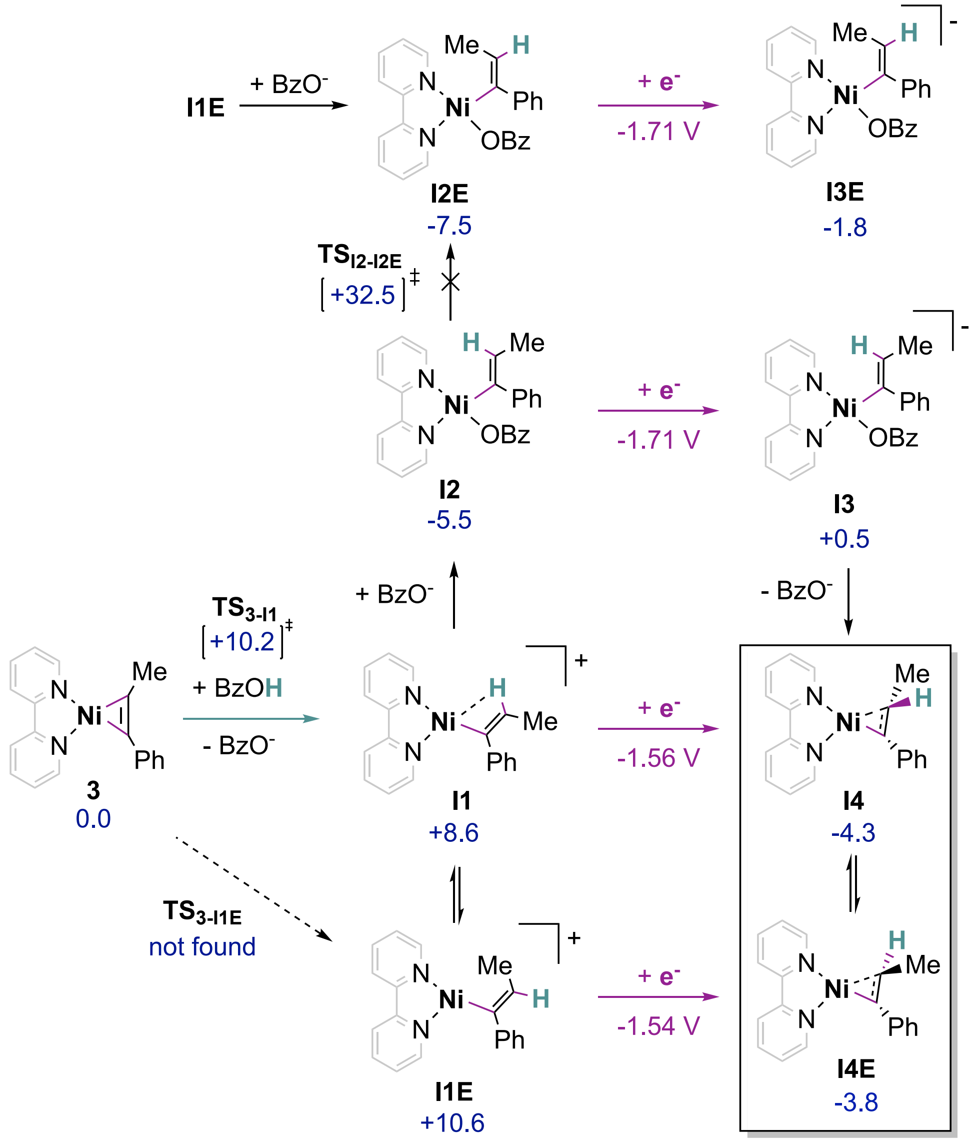

Scheme S4. Gibbs energies for the different isomerization pathways computed at PBE-D3/6-311+G(d,p) level of theory and CPCM model to account for solvent effect (DMF).

SI1 and **SI2** are the two regioisomers of **I1** and **I2** respectively (Scheme S5). These regioisomers were excluded because the stoichiometric protonation of **3** with BzOH (see above SI sections 3.4.2 and 3.4.5) only shows ^{1}H NMR signals corresponding to **I2**.

All the inner-sphere protonation steps of **3, I1 or I4** obtained by computation display a very high barrier (**TS$_{S1}$** to **TS$_{S4}$** > 24 kcal.mol^{-1}).

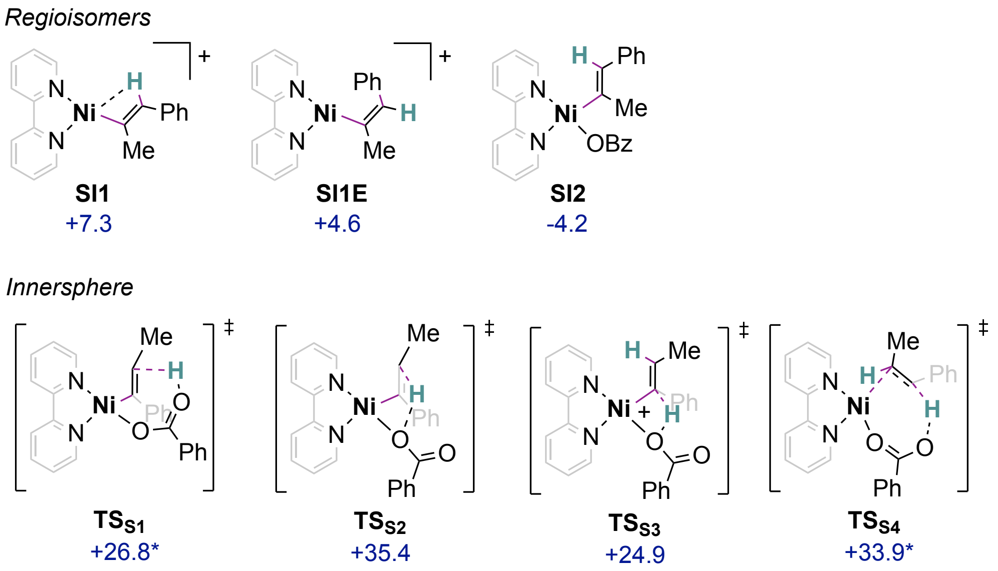

Scheme S5. Computed Gibbs energies for alternative structures and transition states. Conditions: PBE-D3/6-311+G(d,p) level of theory; CPCM model to account for solvent effect (DMF).*Two imaginary frequencies.

5.5. Three lowest frequencies and Gibbs free energy for all computed structures

Structure geometries can be found in the xyz document attached.

H_2

```
      4304.82 cm**-1
Final Gibbs free energy         ...     -1.16807788 Eh
```

Phenazine

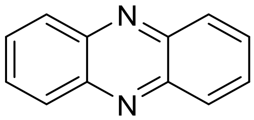

```
        90.89 cm**-1
        96.60 cm**-1
       232.12 cm**-1
Final Gibbs free energy         ...   -570.94722621 Eh
```

Phenazine(•–)

```
        87.87 cm**-1
        90.06 cm**-1
       241.56 cm**-1
Final Gibbs free energy         ...   -571.07091542 Eh
```

BzOH

```
        53.31 cm**-1
       150.29 cm**-1
       210.56 cm**-1
Final Gibbs free energy         ...   -420.39364366 Eh
Final Gibbs free energy         ...   -420.39704840 Eh (BzOD)
```

BzO–

```
            44.23 cm**-1
           151.69 cm**-1
           213.73 cm**-1
Final Gibbs free energy         ...   -419.94487856 Eh
```

S1

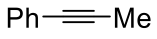

```
            16.90 cm**-1
            70.96 cm**-1
            81.26 cm**-1
Final Gibbs free energy         ...   -347.26863267 Eh
```

(Z)-S1H₂

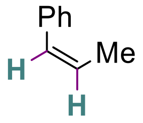

```
            46.61 cm**-1
           125.36 cm**-1
           158.13 cm**-1
Final Gibbs free energy         ...   -348.48054550 Eh
```

(E)-S1H₂

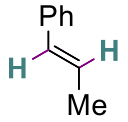

```
            52.41 cm**-1
           122.56 cm**-1
           148.25 cm**-1
Final Gibbs free energy         ...   -348.48484156 Eh
```

1

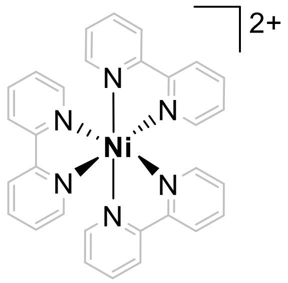

```
            18.35 cm**-1
            23.55 cm**-1
            24.61 cm**-1
Final Gibbs free energy         ...  -2992.28762091 Eh
```

2

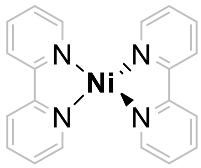


```
          31.85 cm**-1
          35.52 cm**-1
          43.13 cm**-1
Final Gibbs free energy         ...  -2497.73453396 Eh
```

3

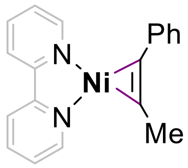


```
          21.17 cm**-1
          25.78 cm**-1
          43.01 cm**-1
Final Gibbs free energy         ...  -2350.20572267 Eh
```

3•– (doublet)

```
          18.33 cm**-1
          25.91 cm**-1
          42.35 cm**-1
Final Gibbs free energy         ...  -2350.30626164 Eh
```

3•– (quadruplet)

```
          11.23 cm**-1
          17.08 cm**-1
          19.51 cm**-1
Final Gibbs free energy         ...  -2350.25287832 Eh
```

4

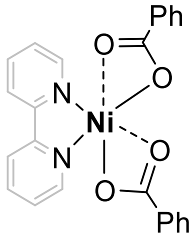


```
          12.72 cm**-1
          19.34 cm**-1
          20.84 cm**-1
Final Gibbs free energy         ...  -2842.54199274 Eh
```

I1

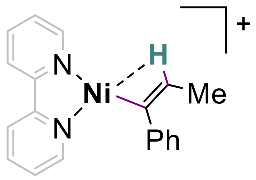


```
          13.04 cm**-1
          28.83 cm**-1
```

```
           36.80 cm**-1
Final Gibbs free energy         ...    -2350.64075659 Eh
```

I1E

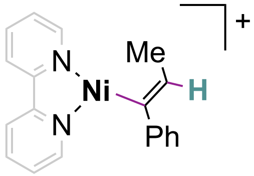

I1E

+10.6

```
           21.50 cm**-1
           24.89 cm**-1
           33.14 cm**-1
Final Gibbs free energy         ...    -2350.63754121 Eh
```

I2

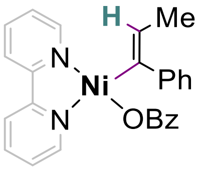


```
           13.93 cm**-1
           19.96 cm**-1
           27.84 cm**-1
Final Gibbs free energy         ...    -2770.60808718 Eh
```

I2E

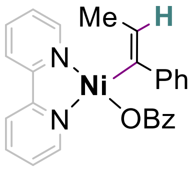


```
            5.74 cm**-1
           21.46 cm**-1
           27.36 cm**-1
Final Gibbs free energy         ...    -2770.61133126 Eh
```

I3

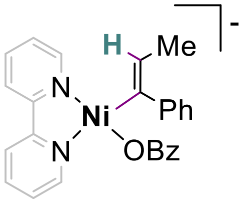


```
           14.52 cm**-1
           21.84 cm**-1
           29.00 cm**-1
Final Gibbs free energy         ...    -2770.71965954 Eh
```

I3E

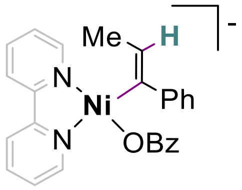

```
           13.10 cm**-1
           23.44 cm**-1
           29.32 cm**-1
Final Gibbs free energy         ...    -2770.72332990 Eh
```

I4

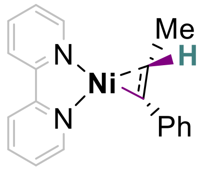

```
           18.92 cm**-1
           31.01 cm**-1
           36.17 cm**-1
Final Gibbs free energy         ...    -2350.77573863 Eh
```

I4E

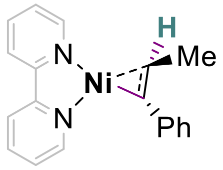

```
           16.08 cm**-1
           29.26 cm**-1
           33.46 cm**-1
Final Gibbs free energy         ...    -2350.77505559 Eh
```

I5

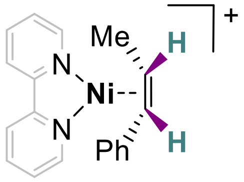

```
            7.49 cm**-1
           10.88 cm**-1
           37.21 cm**-1
Final Gibbs free energy         ...    -2351.24997060 Eh
```

I5E

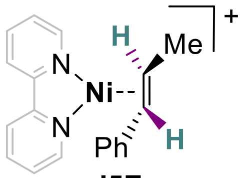

I5E

```
            8.53 cm**-1
           23.13 cm**-1
```

```
            50.54 cm**-1
Final Gibbs free energy          ...   -2351.25723641 Eh
```

I6

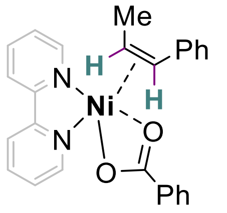


```
            14.56 cm**-1
            20.01 cm**-1
            24.16 cm**-1
Final Gibbs free energy          ...   -2771.21537936 Eh
```

I7

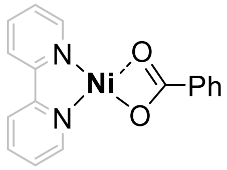


```
            21.82 cm**-1
            24.20 cm**-1
            28.84 cm**-1
Final Gibbs free energy          ...   -2422.73119513 Eh
```

I8

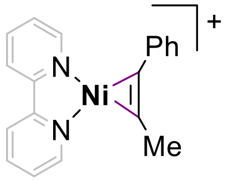


```
            11.01 cm**-1
            14.68 cm**-1
            30.07 cm**-1
Final Gibbs free energy          ...   -2350.04828515 Eh
```

I9

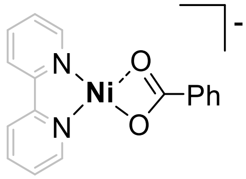


```
            25.64 cm**-1
            28.71 cm**-1
            33.83 cm**-1
Final Gibbs free energy          ...   -2422.83407021 Eh
```

I10

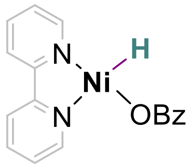


```
          20.47 cm**-1
          23.44 cm**-1
          28.39 cm**-1
Final Gibbs free energy         ...  -2423.30855183 Eh
```

I11

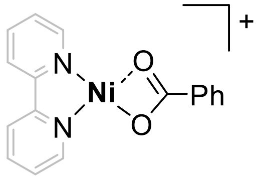


```
          23.84 cm**-1
          26.14 cm**-1
          29.88 cm**-1
Final Gibbs free energy         ...  -2423.12284372 Eh
```

I12

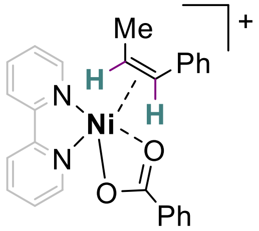


```
          13.23 cm**-1
          18.91 cm**-1
          23.11 cm**-1
Final Gibbs free energy         ...  -2771.05338675 Eh
```

I13

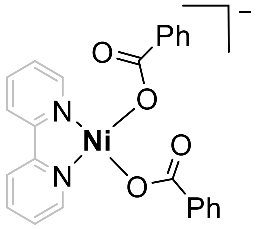


```
           7.83 cm**-1
          10.31 cm**-1
          13.92 cm**-1
Final Gibbs free energy         ...  -2842.67166318 Eh
```

I14

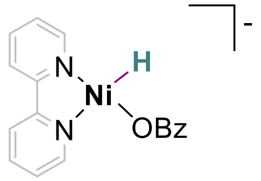

```
          15.15 cm**-1
          19.66 cm**-1
          27.15 cm**-1
Final Gibbs free energy         ...    -2423.42189199 Eh
```

I15

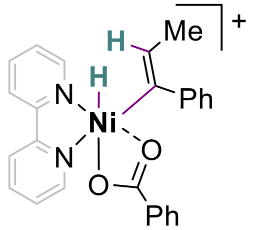

```
           6.76 cm**-1
          20.78 cm**-1
          25.79 cm**-1
Final Gibbs free energy         ...    -2770.99567931 Eh
```

SI1

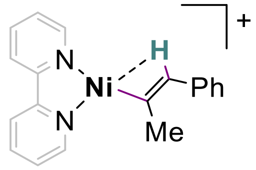

```
          12.42 cm**-1
          24.78 cm**-1
          44.57 cm**-1
Final Gibbs free energy         ...    -2350.64070165 Eh
```

SI1E

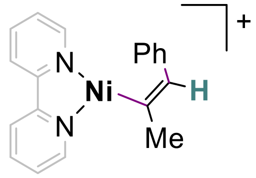

```
          26.51 cm**-1
          39.65 cm**-1
          72.77 cm**-1
Final Gibbs free energy         ...    -2350.64280070 Eh
```

SI2

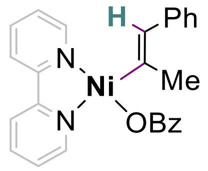

```
          13.02 cm**-1
          19.33 cm**-1
          21.77 cm**-1
Final Gibbs free energy         ...    -2770.60606871 Eh
```

$TS_{3\text{-}I1}$

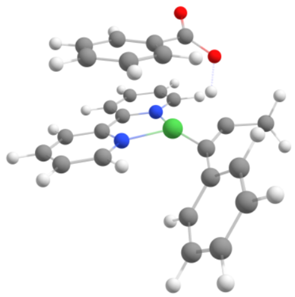

```
        -1086.02 cm**-1 ***imaginary mode***
           11.44 cm**-1
           21.73 cm**-1
Final Gibbs free energy         ...   -2770.58311103 Eh
Final Gibbs free energy         ...   -2770.58470357 Eh(deuterated)
```

$TS_{I3\text{-}I4}$

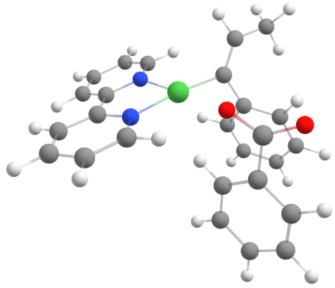

```
          -74.04 cm**-1 ***imaginary mode***
            8.84 cm**-1
           14.57 cm**-1
Final Gibbs free energy         ...   -2770.70357224 Eh
```

$TS_{I5\text{-}I6}$

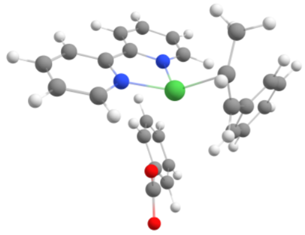

```
          -61.52 cm**-1 ***imaginary mode***
            8.03 cm**-1
           13.99 cm**-1
Final Gibbs free energy         ...   -2771.19214257 Eh
```

$TS_{I9\text{-}I10}$

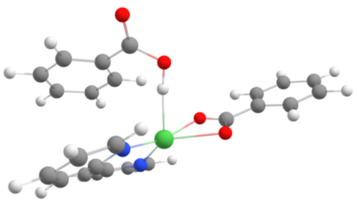

```
          -39.83 cm**-1 ***imaginary mode***
           14.25 cm**-1
           19.98 cm**-1
Final Gibbs free energy         ...    -2843.21780196 Eh
```

TS$_{I10-I11}$

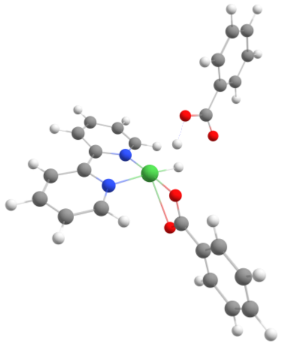

```
         -662.23 cm**-1 ***imaginary mode***
            3.16 cm**-1
           17.65 cm**-1
Final Gibbs free energy         ...    -2843.67018483 Eh
```

TS$_{I10-I2}$

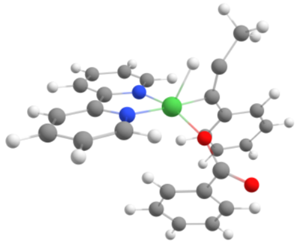

```
         -647.50 cm**-1 ***imaginary mode***
            1.35 cm**-1
           13.09 cm**-1
Final Gibbs free energy         ...    -2770.56581023 Eh
```

TS$_{I2-I12}$

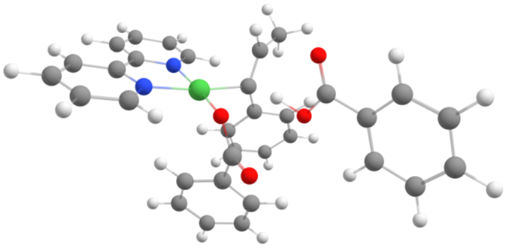

```
         -702.39 cm**-1 ***imaginary mode***
```

```
            1.69 cm**-1
            9.98 cm**-1
Final Gibbs free energy         ...    -3190.96815045 Eh
```

TS$_{I12\text{-}I11}$

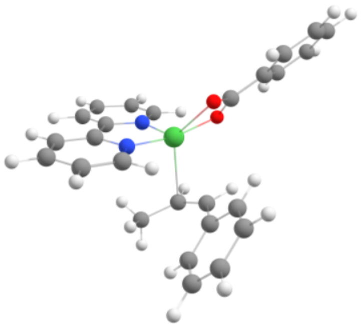

```
          -86.78 cm**-1 ***imaginary mode***
           14.20 cm**-1
           22.73 cm**-1
Final Gibbs free energy         ...    -2771.04104702 Eh
```

TS$_{I13\text{-}I7}$

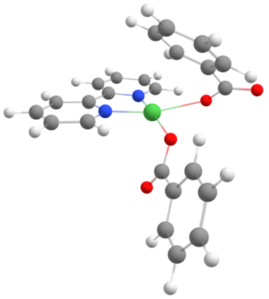

```
          -55.68 cm**-1 ***imaginary mode***
           13.22 cm**-1
           14.95 cm**-1
Final Gibbs free energy         ...    -2842.66447163 Eh
```

TS$_{I14\text{-}I7}$

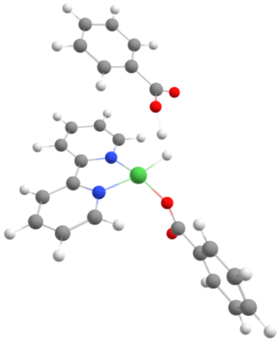

```
        -1087.63 cm**-1 ***imaginary mode***
            3.17 cm**-1
            4.35 cm**-1
Final Gibbs free energy         ...    -2843.80758913 Eh
```

TS$_{I14\text{-}I3}$

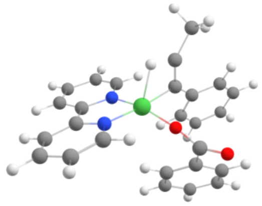

```
          -658.61 cm**-1 ***imaginary mode***
            12.13 cm**-1
            20.26 cm**-1
Final Gibbs free energy         ...    -2770.66923661 Eh
```

TS$_{I2\text{-}I2E}$

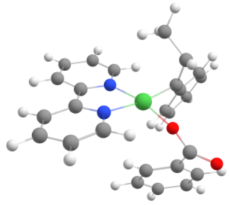

```
           -22.28 cm**-1 ***imaginary mode***
            22.00 cm**-1
            28.55 cm**-1
Final Gibbs free energy         ...    -2770.54751126 Eh
```

TS$_{S1}$

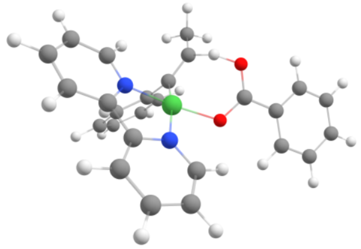

```
          -195.12 cm**-1 ***imaginary mode***
           -21.06 cm**-1 ***imaginary mode***
            13.45 cm**-1
Final Gibbs free energy         ...    -2770.55662593 Eh
```

TS$_{S2}$

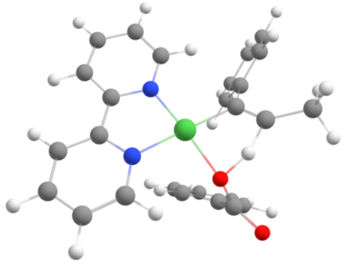

```
           -96.69 cm**-1 ***imaginary mode***
             2.45 cm**-1
            11.67 cm**-1
Final Gibbs free energy         ...    -2770.54299285 Eh
```

TS$_{S3}$

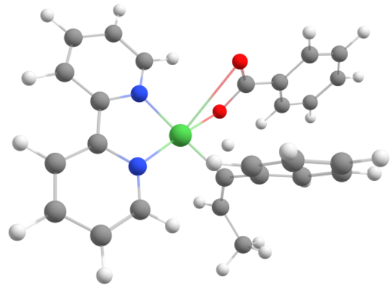

```
        -1179.11 cm**-1 ***imaginary mode***
           10.08 cm**-1
           15.01 cm**-1
Final Gibbs free energy         ...  -2771.00842455 Eh
```

TS$_{S4}$

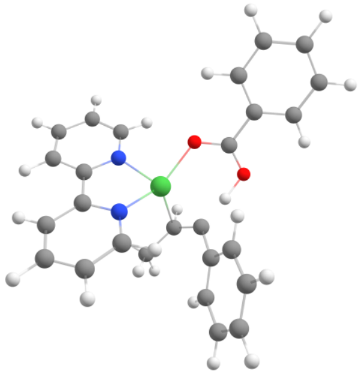

```
         -753.20 cm**-1 ***imaginary mode***
          -72.80 cm**-1 ***imaginary mode***
            7.05 cm**-1
Final Gibbs free energy         ...  -2771.16826173 Eh
```

6. References

1. Hathaway, B. J.; Holah, D. G.; Underhill, A. E., 468. The preparation and properties of some bivalent transition-metal tetrafluoroborate–methyl cyanide complexes. *J. Chem. Soc.* **1962,** (0), 2444-2448.
2. Rosenthal, U.; Nauck, C.; Arndt, P.; Pulst, S.; Baumann, W.; Burlakov, V. V.; Görls, H., Darstellung und eigenschaften des komplexes (dipy)Ni(η2-PhC2SiMe3). Zur korrelation struktureller bindungsparameter mit IR- und NMR-spektroskopischen daten in übergangsmetall-alkin-komplexen. *J. Organomet. Chem.* **1994,** *484* (1), 81-87.
3. Saveant, J. M.; Costentin, C., *Elements of Molecular and Biomolecular Electrochemistry - An Electrochemical Approach to Electron Transfer Chemistry*. Second Edition ed.; John Wiley & Sons, Inc.: 2019.
4. Lee, M. Y.; Kahl, C.; Kaeffer, N.; Leitner, W., Electrocatalytic Semihydrogenation of Alkynes with [Ni(bpy)(3)]2+. *JACS Au* **2022,** *2* (3), 573-578.
5. Schunn, R. A.; Ittel, S. D.; Cushing, M. A.; Baker, R.; Gilbert, R. J.; Madden, D. P., Bis(1,5-Cyclooctadiene)Nickel(0). In *Inorg. Synth.*, 1990; pp 94-98.
6. Day, C. S.; Do, C. D.; Odena, C.; Benet-Buchholz, J.; Xu, L.; Foroutan-Nejad, C.; Hopmann, K. H.; Martin, R., Room-Temperature-Stable Magnesium Electride via Ni(II) Reduction. *J. Am. Chem. Soc.* **2022,** *144* (29), 13109-13117.
7. Baruah, A. M.; Karmakar, A.; Baruah, J. B., Ring opening reactions of pyromellitic dianhydride for the synthesis of first row transition metal dicarboxylate complexes. *Polyhedron* **2007,** *26* (15), 4479-4488.
8. Shen, R.; Chen, T.; Zhao, Y.; Qiu, R.; Zhou, Y.; Yin, S.; Wang, X.; Goto, M.; Han, L. B., Facile regio- and stereoselective hydrometalation of alkynes with a combination of carboxylic acids and group 10 transition metal complexes: selective hydrogenation of alkynes with formic acid. *J. Am. Chem. Soc.* **2011,** *133* (42), 17037-44.
9. Costentin, C.; Drouet, S.; Robert, M.; Savéant, J.-M., Turnover Numbers, Turnover Frequencies, and Overpotential in Molecular Catalysis of Electrochemical Reactions. Cyclic Voltammetry and Preparative-Scale Electrolysis. *J. Am. Chem. Soc.* **2012,** *134* (27), 11235-11242.
10. Fourmond, V.; Jacques, P. A.; Fontecave, M.; Artero, V., H2 evolution and molecular electrocatalysts: determination of overpotentials and effect of homoconjugation. *Inorg. Chem.* **2010,** *49* (22), 10338-47.
11. Neese, F., Software update: The ORCA program system—Version 5.0. *WIREs Computational Molecular Science* **2022,** *12* (5), e1606.

12. Adamo, C.; Barone, V., Toward reliable density functional methods without adjustable parameters: The PBE0 model. *J. Chem. Phys.* **1999,** *110* (13), 6158-6170.
13. Grimme, S.; Ehrlich, S.; Goerigk, L., Effect of the damping function in dispersion corrected density functional theory. *J. Comput. Chem.* **2011,** *32* (7), 1456-1465.
14. Krishnan, R.; Binkley, J. S.; Seeger, R.; Pople, J. A., Self-consistent molecular orbital methods. XX. A basis set for correlated wave functions. *J. Chem. Phys.* **1980,** *72* (1), 650-654.
15. Curtiss, L. A.; McGrath, M. P.; Blaudeau, J. P.; Davis, N. E.; Binning, R. C.; Radom, L., Extension of Gaussian-2 theory to molecules containing third-row atoms Ga–Kr. *J. Chem. Phys.* **1995,** *103* (14), 6104-6113.
16. Clark, T.; Chandrasekhar, J.; Spitznagel, G. W.; Schleyer, P. V. R., Efficient diffuse function-augmented basis sets for anion calculations. III. The 3-21+G basis set for first-row elements, Li–F. *J. Comput. Chem.* **1983,** *4* (3), 294-301.
17. Frisch, M. J.; Pople, J. A.; Binkley, J. S., Self-consistent molecular orbital methods 25. Supplementary functions for Gaussian basis sets. *J. Chem. Phys.* **1984,** *80* (7), 3265-3269.
18. Weigend, F., Accurate Coulomb-fitting basis sets for H to Rn. *Phys. Chem. Chem. Phys.* **2006,** *8* (9), 1057-1065.
19. Cossi, M.; Rega, N.; Scalmani, G.; Barone, V., Energies, structures, and electronic properties of molecules in solution with the C-PCM solvation model. *J. Comput. Chem.* **2003,** *24* (6), 669-681.
20. Moreno, J. J.; Hooe, S. L.; Machan, C. W., DFT Study on the Electrocatalytic Reduction of CO2 to CO by a Molecular Chromium Complex. *Inorg. Chem.* **2021,** *60* (6), 3635-3650.
